# Mechanically reinforced biotubes for arterial replacement and arteriovenous grafting inspired by architectural engineering

**DOI:** 10.1126/sciadv.abl3888

**Published:** 2022-03-16

**Authors:** Dengke Zhi, Quhan Cheng, Adam C. Midgley, Qiuying Zhang, Tingting Wei, Yi Li, Ting Wang, Tengzhi Ma, Muhammad Rafique, Shuang Xia, Yuejuan Cao, Yangchun Li, Jing Li, Yongzhe Che, Meifeng Zhu, Kai Wang, Deling Kong

**Affiliations:** 1 Key Laboratory of Bioactive Materials, Ministry of Education, College of Life Sciences, State Key Laboratory of Medicinal Chemical Biology, Nankai University, Tianjin 300071, China.; 2 Urban Transport Emission Control Research Centre, College of Environmental Science and Engineering, Nankai University, Tianjin 300071, China.; 3 Department of Radiology, Tianjin Key Disciplines of Radiology, Tianjin First Central Hospital, Nankai University, Tianjin 300192, China.; 4 Department of Vascular Surgery, Tianjin Union Medical Center, Nankai University, Tianjin 300121, China.; 5 Department of Ultrasound, Tianjin Union Medical Center, Nankai University, Tianjin 300121, China.; 6 Department of Pathology and Anatomy, School of Medicine, Nankai University, Tianjin 300071, China.; 7 Institute of Transplant Medicine, Tianjin First Central Hospital, Nankai University, Tianjin 300192, China.

## Abstract

There is a lack in clinically-suitable vascular grafts. Biotubes, prepared using in vivo tissue engineering, show potential for vascular regeneration. However, their mechanical strength is typically poor. Inspired by architectural design of steel fiber reinforcement of concrete for tunnel construction, poly(ε-caprolactone) (PCL) fiber skeletons (PSs) were fabricated by melt-spinning and heat treatment. The PSs were subcutaneously embedded to induce the assembly of host cells and extracellular matrix to obtain PS-reinforced biotubes (PBs). Heat-treated medium-fiber-angle PB (hMPB) demonstrated superior performance when evaluated by in vitro mechanical testing and following implantation in rat abdominal artery replacement models. hMPBs were further evaluated in canine peripheral arterial replacement and sheep arteriovenous graft models. Overall, hMPB demonstrated appropriate mechanics, puncture resistance, rapid hemostasis, vascular regeneration, and long-term patency, without incidence of luminal expansion or intimal hyperplasia. These optimized hMPB properties show promise as an alternatives to autologous vessels in clinical applications.

## Teaser

Polymer-reinforced biotubes are optimized for arterial replacement and arteriovenous grafting.

## INTRODUCTION

There is substantial clinical requirement for effective vascular graft alternatives to the patient’s own blood vessels, for use in bypass surgery of coronary and peripheral vascular diseases or for creating arteriovenous graft (AVG; see table S1 for a list of abbreviations) access points for hemodialysis patients. Autologous vessels are the gold standard for vascular repair or creation of arteriovenous fistula, but their availability is complicated by preexisting disease, trauma, anatomic abnormalities, size mismatches, or previous harvesting (*1*–*5*).

Synthetic vascular grafts, such as expanded polytetrafluoroethylene (ePTFE), are clinically used for bridging large-diameter (≥6 mm) vessels and for the construction of AVG for hemodialysis (*3*, *6*, *7*). For the replacement of small-diameter (<6 mm) vessels, such as below-the-knee and coronary artery bypass, synthetic vascular grafts have unacceptably low patency rates due to high incidence of severe intimal hyperplasia (IH) and thrombosis (*3*–*5*, *8*, *9*). Meanwhile, the use of ePTFE for AVG is confronted by several drawbacks, including susceptibility to clotting, infection risk, and inferior primary and secondary patency rates (*1*, *3*, *6*, *7*, *10*–*12*). The above issues are attributable to the lack of tissue regenerative properties and host-graft integration exhibited by commonly used synthetic materials (*9*, *11*, *13*, *14*).

Tissue-engineered vascular grafts (TEVGs) offer a route to readily transplantable blood vessels, capable of promoting tissue regeneration, host-graft integration, and rapid attainment of vascular functionality (*1*, *8*, *15*). TEVG research and development in vitro have achieved promising results. Culturing of autologous fibroblast and endothelial cell (EC) sheets without scaffolds (*10*), decellularized TEVGs obtained from fibroblast-seeded fibrin glue (*7*), and decellularized TEVGs obtained from bioreactor-grown autologous cell–seeded synthetic polymer tubes (*1*, *3*, *6*) have progressed the field of TEVG research and are approaching clinical reality, as demonstrated by the latter’s success in phase 2 clinical trials (*1*, *6*). These TEVGs all exhibited vascular regeneration, high patency, and absence of aneurysm, calcification, or IH (*1*, *3*, *7*) but have prominent caveats due to the limitations of in vitro methodology, such as being time-consuming, having associated contamination risks, and relying on specialized bioreactor culture conditions (*16*–*18*). Previously, an in vivo method of fabricating autologous TEVGs that exploited the foreign body response (FBR) to implanted materials was demonstrated (*18*, *19*). Subcutaneously embedded cylindrical materials stimulated the FBR, driving the production of fibrous capsules surrounding the implants to form traditional biotubes (TBs) (*20*). However, difficulty in maintaining tubular structure and resistance to arterial pressure were encountered, mainly due to the TB having thin walls and insufficient mechanical strength (*20*). Recent attempts to remedy this issue have gained progress. Terazawa *et al.* generated thickened biotubes by using specially designed molds, which resulted in biotubes with improved mechanical properties that performed well as bypass grafts for stenosed arteriovenous shunts but have yet to be assessed for arterial replacement (*21*). In another study, biotube wall thickness was regulated by modifying the FBR to different surface topographies of implanted rods. However, the resultant biotubes underwent dilatation after implantation into porcine carotid arteries, even when structurally supported by poly(ε-caprolactone) (PCL) sheaths (*22*). Sparks fabricated Dacron mesh–reinforced biotubes and pioneered their application in human peripheral arterial bypass, but rates of thrombosis, stenosis occlusion, and aneurysm formation were high (*23*–*25*). These results may be related to insufficient mechanical strength, poor compliance, and incomplete understanding of how structural properties regulate vascular graft mechanical properties.

**Fig. 1.**
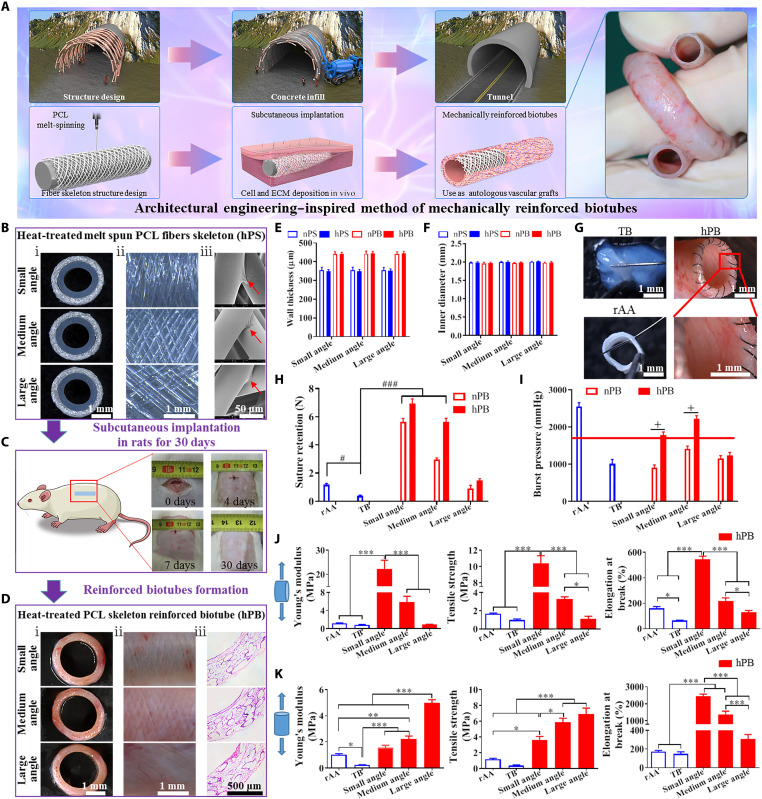
The appearance and physical properties of hPB prepared in the rat subcutis. (**A**) Schematic diagram of the architectural engineering–inspired generation of mechanically reinforced biotubes. (**B**) The morphological characterization of hPS with different fiber winding angles. Stereoscopic images of cross sections (i) and outer surface (ii) of hPS. SEM images showing fiber fusion at crossover points of hPS (iii, red arrow indicates fusion site). (**C**) Process images of dorsum wound healing after SI in rats. (**D**) The morphological characterization of hPB. Stereoscopic images of cross sections (i) and outer surface (ii) of hPB. H&E staining of hPB cross sections showed complete tissue capsule formation in all three kinds of hPB (iii). (**E** and **F**) Quantitative analysis of wall thickness (E) and inner diameter (F) of nPS, hPS, nPB, and hPB based on stereoscopic cross-sectional images (*n* = 5). (**G**) Stereoscopic observation of suturability of TB, hPB, and rAA. (**H**) Quantitative analysis of suture retention (*n* = 5). Statistical significance was calculated by two-way ANOVA with Tukey’s test. The symbol “#” denotes the comparison between different groups, ^#^*P* < 0.05, ^###^*P* < 0.001. (**I**) The burst pressure of rAA, TB, nPB, and hPB (*n* = 5). The red line denotes 1600 mmHg, the threshold for burst pressure of vascular replacements. Statistical significance was calculated by two-way ANOVA with Tukey’s test. The symbol “+” indicates the comparison in the same group, ^+^*P* < 0.05. (**J** and **K**) Quantitative analysis of the radial (J) and axial (K) mechanical properties of rAA, TB, and the hPB with three different fiber winding angles (*n* = 5). Statistical significance was calculated by one-way ANOVA with Tukey’s test. *P < 0.05, **P < 0.01, ***P < 0.001.

Inspired by the architectural design of the steel skeleton used to reinforce concrete for additional strength and stability, PCL fiber skeletons (PSs) were used to provide extra mechanical support to biotubes (Fig. 1A). Previously, blended PCL/collagen electrospun grafts were prepopulated with autologous cells by implant in the rat peritoneal cavity, and the resultant biotubes demonstrated improved patency without dilatation following autologous grafting (*26*). In the present study, a series of PS—the “steel-fiber constructs”—were subsequently embedded to induce the assembly of host cell and extracellular matrix (ECM) in vivo—the “concrete”—forming PS-reinforced biotubes (PBs) with reinforced mechanical strength (Fig. 1A). In contrast to electrospinning, wet-spinning, and freeze-drying techniques, melt-spinning allows the precise control over fiber deposition and mechanical properties of tubular scaffolds while avoiding cytotoxicity concerns associated with solvent use (*27*). We screened PB for optimal use as autologous vascular grafts by assessing the impact of fiber fusion and angle on mechanical and regenerative properties displayed by PB in rat models of abdominal artery replacement. The heat-treated medium-fiber-angle PBs (hMPBs) with superior performance were further evaluated in larger animal models, including canine carotid arterial replacement models (3-mm diameter, 3.5-cm length) and in sheep AVG models (5-mm diameter, 14- to 16-cm length).

## RESULTS

### PB preparation and characterization

PSs with different fiber winding angles (small, medium, and large) were fabricated by melt-spinning. All PSs, including non–heat-treated (nPS) (fig. S1A) and heat-treated (hPS) (Fig. 1B), showed complete wrapping of fibers around medical-grade silicone tubes. Fiber diameters of all PSs were similar with a low degree of variance (≈60 μm) (fig. S2, A and B), irrespective of the differing fiber winding angles (fig. S2C). The PSs were termed as follows: small-angle PS (30.48° ± 1.48°), medium-angle PS (53.81° ± 2.12°), and large-angle PS (111.50° ± 2.77°). Larger pore size correlated with increasing fiber winding angle, and in all PSs, pore sizes were over 100 μm (fig. S2D) to facilitate rapid cell infiltration (*28*, *29*). Total mass of PCL was unaffected by heat treatment but was approximately 0.3 mg/mm less in large-angle PS compared with small-angle PS (fig. S2E). High-magnification scanning electron microscopy (SEM) images showed weak fiber fusion at crossover points in all nPSs (fig. S1A, iii). Heat treatment did not affect PCL fiber wrapping on silicone tubes (Fig. 1B, i) but did exhibit improved fiber fusion at crossover points in all hPSs (Fig. 1B, iii). In addition, the fiber diameter, pore size, and fiber winding angle did not differ between nPS and hPS (fig. S2, B to D). Throughout this article, hPS abbreviations are prefixed according to fiber winding angles where appropriate: small-angle hPS, medium-angle hPS (hMPS), and large-angle hPS; nPSs are prefixed according to fiber winding angles where appropriate: small-angle nPS, medium-angle nPS, and large-angle nPS.

All PSs were implanted into the rat dorsal subcutaneous pouch to evaluate their capability to form PB. Incision wounds healed quickly after subcutaneous implantation (SI), and no swelling or adverse reactions occurred at the implantation sites (Fig. 1C). During SI, the body temperature and weight of rats in the implantation group remained comparable to those in the sham group and those in the normal group (without operation) (fig. S3). Throughout this article, nPS/hPS-reinforced biotube (nPB/hPB) abbreviations are prefixed according to fiber winding angles where appropriate: small-angle nPB/hPB, medium-angle nPB/hMPB, and large-angle nPB/hPB. After SI for 30 days, stereomicroscopy and hematoxylin and eosin (H&E) analysis showed that the pores of all types of PS (Fig. 1D and fig. S1B) had been filled with the host’s tissue and cells. By comparison of the outer surface of PS (Fig. 1B, ii, and fig. S1A, ii) to PB (Fig. 1D, ii, and fig. S1B, ii), we observed that all types of PS maintained the original fiber structure following SI for 30 days. The wall thickness of each type of nPB and hPB was approximately 440 μm, which showed an increase of approximately 90 μm compared to the original nPS or hPS before SI (Fig. 1E). The internal diameter of each type of nPB and hPB showed negligible change (Fig. 1F).

Following SI of medical-grade silicone rods in rats for 30 days, TBs without polymer skeleton supports were obtained. TB failed to maintain tubular structure, and the ends of the TB were difficult to distinguish, which, in turn, increased suture difficulty (Fig. 1G). All types of hPB effectively maintained tubular structure and exhibited similar suturability properties to rat abdominal artery (rAA), without incidence of fiber fraying (Fig. 1G). The suture retention strength of TB was significantly lower than that of rAA. The incorporation of PS, with the exception of large-angle PS, significantly increased suture retention strength of PB beyond that of rAA (Fig. 1H). The burst pressures of the TB and the three types of nPB were below 1600 mmHg, indicating a risk of rupture if they were used as arterial grafts. Heat treatment increased burst pressure thresholds, as demonstrated by small-angle hPB (1780.00 ± 84.49 mmHg) and hMPB (2212.00 ± 92.30 mmHg). However, large-angle hPB demonstrated moderate benefit to burst pressure (below 1600 mmHg) (Fig. 1I). On the basis of burst pressure data, we excluded all types of nPB and only further evaluated hPB as candidate grafts. In the case of small-angle hPB, the circumferentially wrapped fibers were dense and lacked radial elasticity (fig. S4). In the case of large-angle hPB, the axially wrapped fibers were dense and lacked axial elasticity, which resulted in kink formation upon folding at 180° (fig. S4). The hMPB had good elasticity properties in both radial and axial directions, which resulted in facile restoration of original shape after pressing, propping, and stretching, and maintained tube patency without kinking when folded at 180° (fig. S4). Comprehensive analysis of the radial and axial mechanical properties (Young’s modulus, tensile stress, and elongation at break) revealed that the mechanical properties of hMPB had the closest resemblance to rAA (Fig. 1, J and K). These results indicated that hMPB had the optimal mechanical properties as a candidate graft.

### Screening of hPB in rat models

**Fig. 2.**
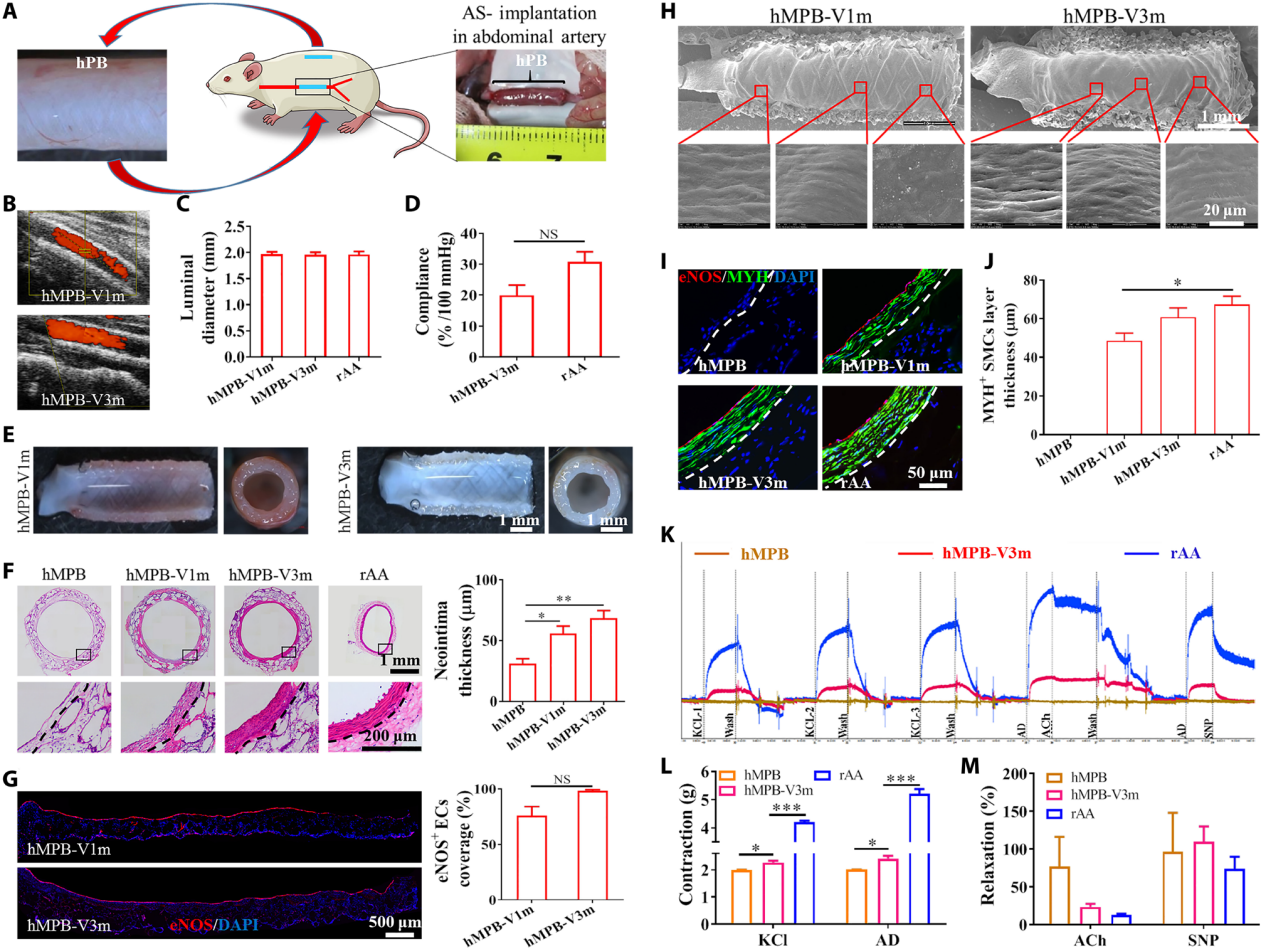
Evaluation of the potential of hMPB as autologous grafts in rAA replacement model. (**A**) Subcutaneously prepared hPBs were used as autologous abdominal artery replacements in rats. (**B**) CDU images of hMPB-V1m and hMPB-V3m and (**C**) corresponding measurement of luminal diameter. (**D**) Quantification of in vivo compliance of implanted hMPB and rAA. (**E**) Lumen and enface views of hMPB-V1m and hMPB-V3m. (**F**) H&E images of hMPB-V1m, hMPB-V3m, and rAA cross sections; quantification of hMPB neointima thickness shown alongside. Black dashed lines represent PS wall boundary (hMPB, hMPB-V1m, and hMPB-V3m) or boundary between tunicae media and adventitia layers (rAA). (**G**) EC coverage was visualized and calculated on the basis of anti-eNOS IF staining of hMPB longitudinal sections before and after VI. (**H**) EC coverage of lumen surfaces of hMPB-V1m and hMPB-V3m observed by SEM. (**I**) Co-stained cross sections show the distribution of ECs (eNOS, red) and SMCs (MYH, green) in hMPB-V1m, hMPB-V3m, and rAA. White dashed lines represent PS wall boundary (hMPB, hMPB-V1m, and hMPB-V3m) or the boundary between tunicae media and adventitia layers (rAA). (**J**) MYH^+^ SMC layer thickness calculated from eNOS/MYH co-staining. (**K**) Representative curves of physiological functions of hMPB, hMPB-V3m, and rAA in response to vasodilators and vasoconstrictors. (**L**) Quantification of constriction in response to KCl and AD; (**M**) Quantification of relaxation in response to ACh and SNP. Samples were preconstricted with AD, and then responses to vasodilators were assessed. Data are representative of *n* = 5 rats. Unpaired Student’s *t* test (D and G) and one-way ANOVA followed by Tukey’s test (C, F, J, L, and M) were performed. *P < 0.05, **P < 0.01, ***P < 0.001. NS, not significant.

We auto-implanted the different angled hPB into rAA to evaluate their regenerative characteristics (Fig. 2A, *n* = 5). TBs were used as baseline controls (*n* = 3). The TB lacked the ability to maintain tubular shape, which increased suture difficulty. The average suture time of TB (91.30 ± 14.20 min) was approximately double that of hPB (40.80 ± 6.40 min). At 3 days after vascular implantation (VI), one of the three rats died from rupture of the implanted TB (fig. S5, A and B). Uneven needle spacing and wrinkling were also problems encountered with TB suturing (fig. S5A). Color Doppler ultrasound (CDU) testing showed that two other TBs used for VI were dilated and exhibited blood reflux at 7 days, and these were further aggravated at 1 month (fig. S5, C and E). H&E images showed that there was serious IH (fig. S5D) or inversion (fig. S5F) at the suture end. The occlusion and stenosis rates of small-angle hPB at 1 month after VI (hPB-V1m) were 20 and 40%, respectively (fig. S6, A to C). The average lumen area of cross sections taken from small-angle hPB-V1m (1.85 ± 0.50 mm^2^) was significantly reduced from pre-VI small-angle hPB (3.06 ± 0.10 mm^2^) (fig. S6D), which reflected the high incidence of IH observed in small-angle hPB-V1m. During the 1 month of VI in the large-angle hPB group, four of the five rats died from severe graft rupture, and the surviving rat exhibited graft dilatation (fig. S7). Satisfactorily, after VI for 1 and 3 months, all hMPB showed patency without stenosis and dilatation (Fig. 2B), and the luminal diameter of hMPB remained unchanged, as assessed by CDU (Fig. 2C). After 3 months of VI, hMPB showed pulsation with changes in blood pressure (movie S1), and compliance reached 19.85 ± 3.14%/100 mmHg; this value was closer to rAA (Fig. 2D). The lumen of hMPB after VI for 1 month (hMPB-V1m) and 3 months (hMPB-V3m), as observed by stereomicroscopy, was free of thrombi (Fig. 2E). The transverse H&E images showed that smooth tissue layers had covered the lumen of hMPB. With extended VI time, the neointima thickness gradually increased and, by 3 months, had formed tissue with a similar tissue structure to that observed in tunicae media of rAA (Fig. 2F). Next, we used anti–endothelial nitric oxide synthase (eNOS) antibody to detect the regeneration of functional ECs. There were no eNOS^+^ ECs on the lumen of hMPB (Fig. 2G). After VI for 1 month, eNOS^+^ EC coverage rate in hMPB reached 76.00 ± 3.09%. At 3 months, the lumens of hMPB were completely covered by eNOS^+^ ECs (Fig. 2G). SEM images also confirmed that EC coverage increased with increasing VI time, and regenerated ECs exhibited cobblestone-like morphology, elongated in the direction of blood flow (Fig. 2H). The smooth muscle myosin heavy chain I (MYH) and eNOS immunofluorescence (IF) staining showed that no MYH^+^ contractile smooth muscle cells (SMCs) could be observed in the initial hMPB (Fig. 2I). However, an MYH^+^ SMC layer was evident at 1 month after VI and had an average thickness above 48.54 ± 4.05 μm, and this increased to 60.39 ± 4.47 μm by 3 months, comparable to the thickness of the MYH^+^ SMC layer in rAA (Fig. 2J). Co-stained images indicated that eNOS^+^ functional ECs and MYH^+^ contractile SMCs closely adhered to each other throughout the regeneration process (Fig. 2I). The tissue structure of hMPB-V3m was consistent with that of rAA. Vascular physiological functional testing indicated that hMPB before VI had no physiological activity (Fig. 2K). At 3 months after VI, the hMPB displayed the vascular functionalities of contraction and relaxation (Fig. 2K). hMPB-V3m constricted in response to the vasomotor agonists, potassium chloride (KCl) and adrenaline (AD), although the magnitude of constriction was significantly less than that of rAA (Fig. 2L). hMPB-V3m also displayed vasodilation to endothelium-independent vasodilators including endothelial-specific activator acetylcholine (ACh) and sodium nitroprusside (SNP) (Fig. 2M), suggesting a regulatory involvement between the regenerated ECs and contractile SMCs. These results indicated that hMPB had the superior capacity for vascular regeneration and host-graft integration in rAA replacement models.

### The availability of hMPB in different species

**Fig. 3.**
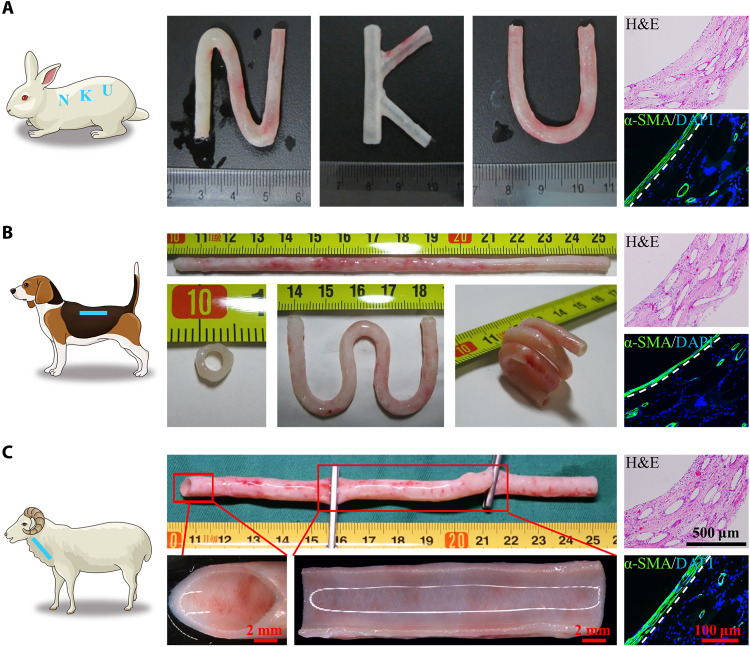
The availability of hMPB in different species at 30 days after SI. (**A**) Tailor-made hMPB could be fabricated in subcutaneous space of rabbit. (**B**) hMPB prepared in subcutaneous space of canine had excellent flexibility. (**C**) hMPB prepared in subcutaneous space of sheep still maintained original skeleton structure despite oblique cutting, clamping, and twisting. H&E staining of cross sections showed that subcutaneously implanted hMPS in rats, canine, and sheep have been filled with autologous cells and tissue at 30 days after operation. IF staining with anti–α-SMA antibody showed that the lumen surface of hMPB prepared in different species were all covered by a thin layer of α-SMA^+^ cell with circumferential alignment, and microvasculature was present throughout hMPB. The white dashed lines represent the boundary between inner α-SMA^+^ cell tissue layer and hMPS wall in the hMPB.

After SI for 30 days, hMPB could be successfully prepared in rabbit, canine, and sheep (Fig. 3). All hMPSs were filled with host tissue and cells, as evidenced by H&E analysis. The lumens showed thin and circumferentially aligned alpha smooth muscle actin^+^ (α-SMA^+^) cell layers, and α-SMA^+^ microvasculature was detectable throughout the hMPB, as evidenced by IF staining. These results were consistent with hMPB prepared in rat. By modifying the design and shape of the hPS, customizable hMPBs were obtained from rabbits (Fig. 3A), indicating the applicability of hMPB to personalized medicine. hMPBs obtained from canine have no branches or valves and displayed a high degree of flexibility. The length of hMPB was approximately 15 cm in canine (Fig. 3B) and could be increased further to 50 cm in sheep (fig. S8), demonstrating the feasible size scalability to meet clinical requirements. Despite oblique cutting, clamping, or twisting, the hMPB maintained the original shape of the hMPS structure and lumen smoothness, without fiber fraying (Fig. 3C).

### Performance of hMPB in canine models of peripheral arterial replacement

**Fig. 4.**
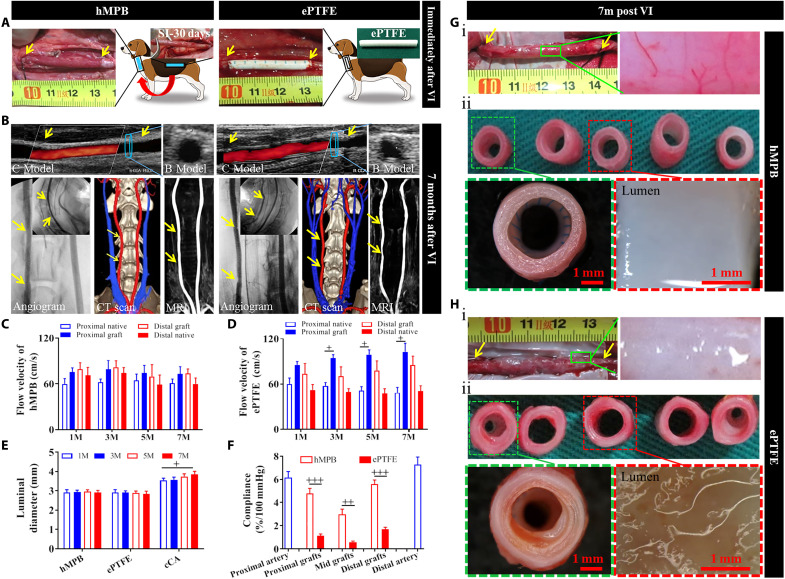
hMPB and ePTFE graft implantation in cCA replacement model. (**A**) hMPB and ePTFE graft images before VI and immediately after VI (*n* = 3). (**B**) Representative CDU, angiography, CT 3D reconstruction, and MRI images of hMPB and ePTFE grafts at 7 months after VI (yellow arrows indicate suture sites). (**C** to **E**) Ultrasound-based measurement of flow velocity (C and D) and luminal diameter (E) of hMPB and ePTFE grafts after VI (*n* = 3). Statistical significance was calculated by two-way ANOVA with Tukey’s test. Symbol (+) indicates the comparison in the same group, ^+^*P* < 0.05. (**F**) Quantification of in vivo compliance of the hMPB-V7m and ePTFE-V7m as well as their adjacent proximal and distal cCA (*n* = 3). Statistical significance was calculated by two-way ANOVA with Tukey’s test. Symbol (+) indicates the comparison in the same group, ^++^*P* < 0.01, ^+++^*P* < 0.001. (**G**) hMPB-V7m and (**H**) ePTFE-V7m exterior view (i) and five cross-sectional pieces (ii). Green boxes indicate zoomed exterior views. Green dashed boxes indicate sutured end and magnified view. Red dashed boxes indicate the pieces taken from the middle of the graft for observation of graft lumens.

The hMPBs prepared in subcutis of canines were sutured as autologous interposition grafts into the canine carotid artery (cCA), and ePTFE grafts were used as control grafts (Fig. 4, yellow arrow indicates sutured sites). After restoration of blood flow, the two types of grafts showed no bleeding (Fig. 4A). CDU, angiography, computed tomography (CT), and magnetic resonance imaging (MRI) of hMPB and ePTFE grafts at 7 months after VI (hMPB-V7m and ePTFE-V7m) showed that the two types of grafts were patent without dilatation. hMPBs were absent of stenosis (Fig. 4B) and showed comparable blood flow velocity with the adjacent cCA throughout the entire VI period (Fig. 4C). However, ePTFE grafts showed obvious stenosis at the proximal sutured ends (Fig. 4B) and a significantly increased blood flow velocity (Fig. 4D). After VI, the luminal diameter (Fig. 4E) of hMPB and ePTFE grafts showed no changes during the 7 months, whereas the luminal diameter of cCA increased by 10.42% (1 month diameter, 3.55 ± 0.11 mm; 7 months diameter, 3.92 ± 0.09 mm; *P*<0.05) (Fig. 4E). At 7 months after VI, hMPB showed good compliance, while ePTFE grafts displayed limited pulsation with changes in blood pressure (movie S2). The compliance values of hMPB-V7m at the proximal, middle, and distal positions were 4.77 ± 0.45%/100 mmHg, 2.97 ± 0.48%/100 mmHg, and 5.61 ± 0.51%/100 mmHg, respectively. These values were all lower than the cCA but were significantly higher than the compliance values of the corresponding positions in ePTFE-V7m grafts (Fig. 4F). Angiography images showed that hMPB could bend with the twisting of the neck, while the ePTFE grafts maintained their rigid state at 7 months. Three-dimensional (3D)–CT and MRI images showed that compared to ePTFE grafts, hMPBs have curvature more closely resembling the contralateral cCA at 7 months (Fig. 4B). These results indicated that hMPB had good flexibility and compliance in vivo. After VI for 7 months, hMPB was easily separated from adhered surrounding tissue and some microvasculature had formed to surround the hMPB, indicating that hMPB had well integrated with the host tissue (Fig. 4G, i). However, the separation of ePTFE grafts was difficult, and nonvascularized adhered fibrous capsules were obvious at 7 months (Fig. 4H, i). The explanted grafts were divided into five pieces and observed by stereomicroscopy (Fig. 4G, ii, and H, ii). The ePFTE grafts showed stenosis at both sutured ends and coagulation component–rich lumens (Fig. 4H, ii). Satisfactorily, all five pieces of the hMPB-V7m were clear of stenosis, with smooth lumens without thrombus formation (Fig. 4G, ii).

### Remodeling of hMPB in canine

**Fig. 5.**
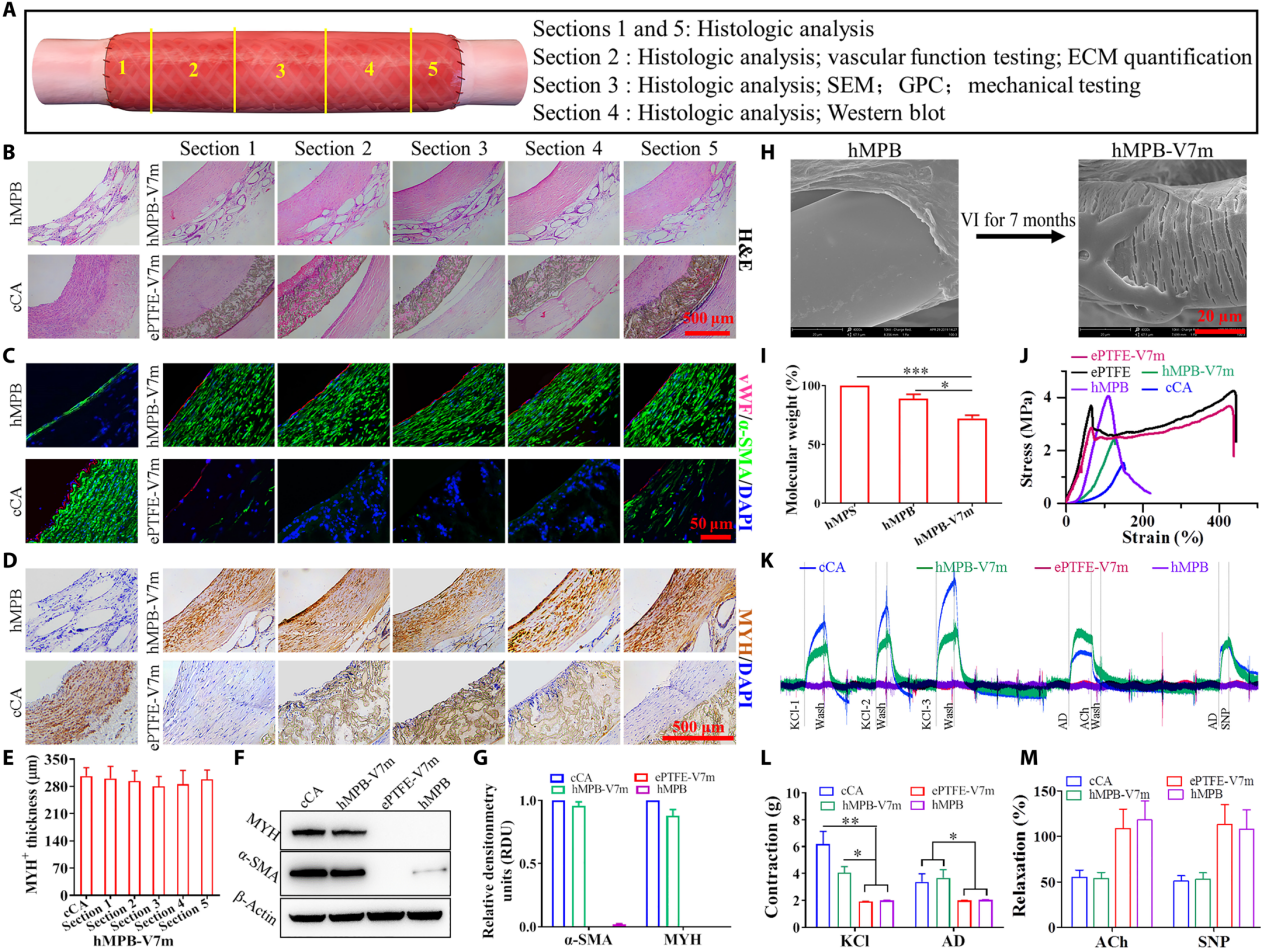
hMPB and ePTFE grafts remodeling at 7 months after VI into cCA. (**A**) Schematic diagram of corresponding analysis for five vessel samples of hMPB-V7m and ePTFE-V7m. (**B**) H&E staining of cross sections. (**C**) Co-IF staining images with vWF and α-SMA antibody. (**D**) The contractile SMCs were visualized using IHC staining with MYH antibody. (**E**) Quantification of contractile SMCs thickness based on MYH IHC staining images of cCA and hMPB-V7m (*n* = 3). (**F**) WB analysis of α-SMA and MYH protein expression of corresponding samples. (**G**) Quantification of α-SMA and MYH protein from the WB analysis (*n* = 3). (**H**) SEM images showed the degradation of hMPS within hMPB-V7m. (**I**) Molecular weights analysis of hMPS and PCL from extracts of hMPB and hMPB-V7m by GPC (*n* = 3). Statistical significance was calculated by one-way ANOVA with Tukey’s test. *P < 0.05, ***P < 0.001. (**J**) Stress-strain curves of corresponding samples. (**K**) Representative curves of physiological functions of corresponding samples displaying different degrees of sensitivity to vasodilators and vasoconstrictors. (**L**) Quantification of constriction in response to KCl and AD (*n* = 3). Statistical significance was calculated by one-way ANOVA with Tukey’s test. *P < 0.05, **P < 0.01. (**M**) Quantification of relaxation in response to ACh and SNP (*n* = 3).

Each of the five pieces of the hMPB-V7m and ePTFE-V7m from canine models were analyzed as indicated (Fig. 5A). Macroscopic cross-sectional H&E images showed that the hMPB-V7m had formed homogeneous neointima without any fibrous capsule, whereas obvious IH at both suture ends, thrombi, and extensive fibrous capsules were observed in ePTFE-V7m (fig. S9). High-magnification H&E showed that neointima formed in hMPB-V7m was similar to tunica media of cCA (Fig. 5B). The hMPS within hMPB before and after VI for 7 months maintained complete cellularization, while few cells had infiltrated into the ePTFE graft wall at 7 months, and no cells were observed in the fibrous capsules surrounding ePTFE grafts (Fig. 5B). Co-staining with von Willebrand Factor (vWF) and α-SMA antibodies (Fig. 5C) showed that after VI for 7 months, the lumen of hMPB was completely covered by vWF^+^ EC monolayer, and circumferentially aligned α-SMA^+^ SMC layers closely adhered to the ECs, similar to cCA cell arrangement. The lumen of ePTFE grafts had few vWF^+^ ECs and displayed randomly arranged α-SMA^+^ SMCs at both sutured ends at 7 months. SEM images showed that regenerated ECs in hMPB-V7m exhibited cobblestone-like morphology with a nice elongation in the direction of blood flow (fig. S10), similar to regenerated ECs in the rat model. In contrast, coagulation matrix adhered on the lumen surface of ePTFE-V7m, and no clear ECs morphology was observable (fig. S10). The regeneration of MYH^+^ contractile SMCs in hMPB was detected (Fig. 5D). hMPB-V7m formed a complete MYH^+^ SMC layer, with the thickness comparable to cCA (Fig. 5E). However, no MYH^+^ SMCs were observed in ePTFE-V7m (Fig. 5D). Western blot (WB) analysis indicated that α-SMA and MYH protein expression of hMPB-V7m was similar to cCA, while α-SMA protein expression was limited in hMPB and no MYH protein expression was detected in hMPB or ePTFE-V7m (Fig. 5, F and G). Compared to hMPB, hMPS within hMPB-V7m had disintegrated (Fig. 5H). Gel permeation chromatography (GPC) analysis revealed that the molecular weight of hMPS decreased to 88.72% in hMPB after 30 days of SI and then further decreased to 72.05% in hMPB-V7m (Fig. 5I). Stress-strain curve showed that the mechanical properties of hMPB-V7m decreased compared to pre-VI hMPB but attained a “J-shaped” curve resembling cCA (Fig. 5J). However, ePTFE grafts, before and after VI, did not exhibit J-shaped curves and showed no obvious changes in mechanical properties (Fig. 5J). hMPB-V7m had physiological activity, which included constriction in response to vasomotor agonists (KCl and AD) and relaxation in response to endothelium-independent vasodilators (ACh and SNP). However, the hMPB and ePTFE-V7m demonstrated no physiological activity in response to the above stimuli (Fig. 5K). Quantitative analysis showed that the contraction of hMPB-V7m in response to KCl and AD (Fig. 5L) and the relaxation of hMPB-V7m in response to ACh and SNP (Fig. 5M) were closer to the responses measured in cCA.

### Evaluation of the performance of hMPB as AVG in sheep models

**Fig. 6.**
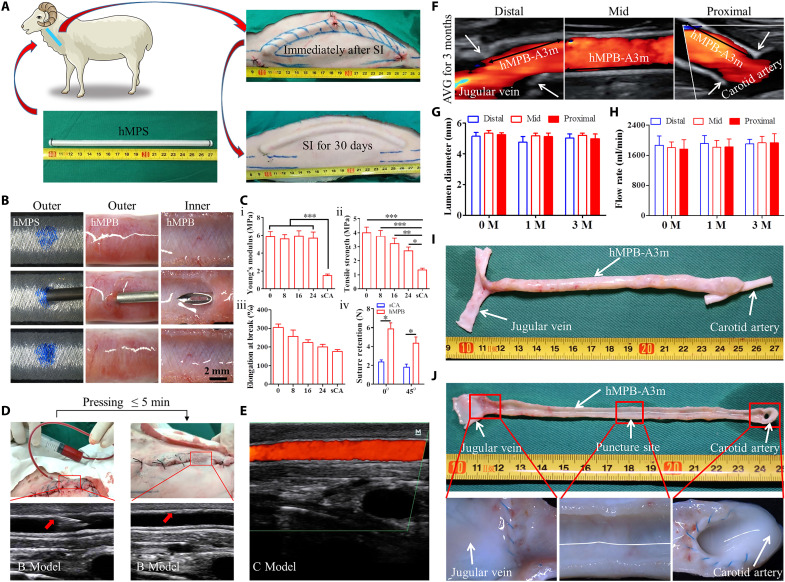
hMPB implantation in a sheep AVG model. (**A**) Subcutaneous implant of hMPS in sheep neck according to prepositioned tunnel to prepare hMPB connecting the carotid artery and jugular vein (*n* = 3). (**B**) Macroscopic observations before and after 16G hemodialysis needle punctures. (**C**) Quantitative analysis of hMPB mechanical properties after 0, 8, 16, and 24 punctures (i to iii). Straight-across (0°) and oblique (45°) suture retention of hMPB and sCA were assessed and statistically analyzed (iv) (*n* = 5). (**D**) Macroscopic observation and CDU showed that after hMPB autografts as AVG, the hMPB segment still integrated with subcutaneous tissue, allowed for immediate needle puncture, and achieved hemostasis with 5 min of applied pressure. (**E**) C-model color ultrasound showed that no blood flowed from puncture sites after pressing. (**F**) Representative ultrasound images of hMPB as AVG in sheep at 3 months (hMPB-A3m). (**G** and **H**) Ultrasound-based measurement of proximal, middle, and distal diameter of hMPB (G) and flow rate (H) over the course of implantation (*n* = 3). (**I**) Explant exterior at 3 months showing loose connective tissue and microvasculature formation. (**J**) Low-magnification (top) and high-magnification (bottom) lumen view at 3 months. Top panel showed that the entirety of the hMPB lumen was free of thrombus formation; bottom panels showed absence of stenosis at sutured ends and no obvious changes at puncture site. One-way ANOVA followed by Tukey’s tests (C, i to iii, G, and H) and unpaired Student’s *t* test (C, iv) were performed; significance indicated by *P < 0.05, **P < 0.01, ***P < 0.001.

The performance of hMPB as AVG was evaluated in sheep models. hMPSs combined with silicone tubes cores were implanted subcutaneously into the neck of sheep at the site of the predetermined AVG tunnel carotid artery and jugular vein (Fig. 6A). After 30 days, the hMPB (5 mm diameter) formed in position. hMPS and hMPB could quickly restore the original structure after puncture with a 16G dialysis needle (Fig. 6B). After puncture counts of 0, 8, 16, and 24 times (according to ISO 7198:2016), radial mechanical testing showed that the Young’s modulus (Fig. 6C, i) of hMPB exhibited almost no significant change; however, tensile stress (Fig. 6C, ii) and elongation (Fig. 6C, iii) at break showed reductions with accumulative number of punctures, but these measurements remained higher than those taken from sheep carotid artery (sCA) (Fig. 6C, i to iii). End-to-side anastomosis is often used in clinical application of AVG. To achieve end-to-side anastomosis, oblique cuts were made at the two ends of hMPB grafts. The suture retention measurements of straight-across (0°) and oblique end (45°) of hMPB were 5.23 ± 0.79 N and 4.38 ± 1.09 N, respectively, which were significantly higher than those of corresponding sCA (Fig. 6C, iv). The cut ends of the hMPB that were adjacent to the carotid artery and jugular vein were separated from the surrounding tissue (separation region) before the hMPB was anastomosed to carotid artery and jugular vein in an end-to-side manner. The middle of hMPB that was not separated from the surrounding tissue was named integration region. The in vivo puncture resistance of hMPB was immediately tested using 16G dialysis needles after operation. hMPB demonstrated good in vivo puncture resistance regardless of separation region (fig. S11) or integration region (Fig. 6D), as evidenced by CDU analysis and the achievement of hemostasis within 5 min of applied pressure at the puncture site (Fig. 6D and fig. S11). Time to hemostasis after compression was less than 5 min in both regions. Further CDU testing revealed that the puncture did not induce tubular wall damage or blood leakage from hMPB (Fig. 6E). Subsequently, puncture testing and ultrasound were performed once a month (fig. S12). Throughout the 3-month AVG period, ultrasound testing showed that hMPB remained patent (Fig. 6F) and maintained an approximately 5-mm diameter (Fig. 6G) and 1800 ml/min flow rate (Fig. 6H). At 3 months after AVG, stereomicroscopy showed that hMPB still had good tubular structure without dilatation or rupture (Fig. 6I) and smooth luminal surfaces without thrombus formation or incidence of IH (Fig. 6J). Furthermore, high-magnification stereomicroscopy showed that hMPBs were well integrated with the carotid artery and jugular vein at the two sutured ends and could effectively resist puncture without exhibiting obvious changes at puncture site (Fig. 6J).

### Remodeling of hMPB as AVG in sheep

**Fig. 7.**
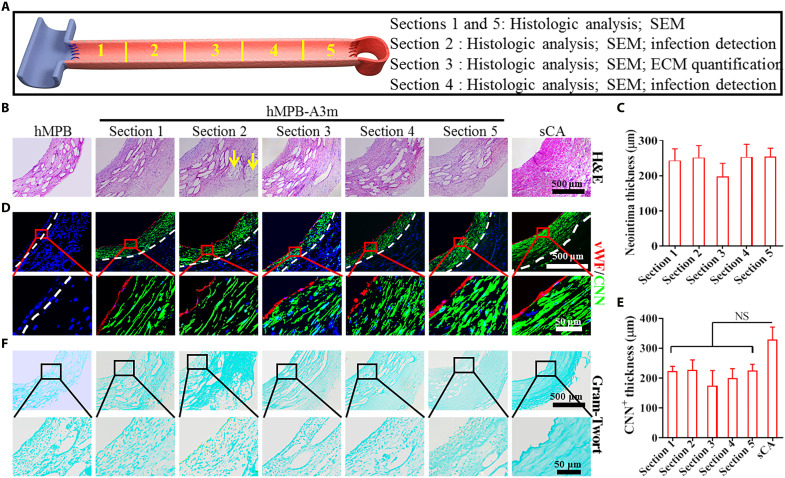
The histological analysis and infection detection of hMPB as AVG for 3 months in the sheep model. (**A**) Schematic diagram of corresponding analysis of five sample sections of hMPB as AVG after implantation between jugular vein and carotid artery for 3 months. (**B**) H&E staining images of cross sections. (**C**) Quantitative analysis of neointima thickness of hMPB-A3m based on H&E images (*n* = 3). Statistical significance was calculated by one-way ANOVA with Tukey’s test. (**D**) The regeneration of ECs and contractile SMCs was observed by Co-IF staining with vWF and CNN. The white dashed lines represent the PS wall boundary (hMPB and hMPB-A3m) or the boundary between tunicae media and adventitia layers (sCA). Bottom-layer images represent the high magnification of the red area within top-layer images. (**E**) The thickness of CNN^+^ was calculated on the basis of low-magnification (top layer) co-IF staining images (*n* = 3). Statistical significance was calculated by one-way ANOVA with Tukey’s test. NS indicates not significant. (**F**) Gram-Twort staining showed no bacteria survival in hMPB, hMPB-A3m, and sCA. Bottom-layer images represent high magnification of the black area within top-layer images.

hMPBs 3 months after AVG (hMPB-A3m) in sheep models were obtained and analyzed as indicated (Fig. 7A). H&E images showed that compared to hMPB, hMPB-A3m formed an obvious neointima that resembled the tunica media of sCA (Fig. 7B). The neointima thickness of the hMPB-A3m sample pieces was comparable at approximately 230 μm (Fig. 7C). An abundance of cells and some adipose tissue (yellow arrows) (Fig. 7B) was observed in hMPB-A3m, which indicated the integration of the outer tissue interface with the subcutaneous tissue. Co-staining for the EC marker, vWF, and the contractile SMC marker, calponin (CNN), showed that before AVG implantation, no ECs or contractile SMCs were detected in hMPB, whereas, at 3 months after AVG, the layer of circumferentially aligned CNN^+^ SMCs had been formed and was completely covered by vWF^+^ ECs (Fig. 7D). The thickness of CNN^+^ SMCs was approximately 66% of the sCA SMC layer (Fig. 7E). The regenerated and closely adhered ECs and SMCs were analogous in their cell arrangement to sCA tissue. SEM images showed that regenerated ECs in hMPB-A3m exhibited cobblestone-like morphology with elongation in the direction of blood flow (fig. S13), which mirrored observations made in rat and canine models. Our assessment of bacterial infection risk revealed that hMPB-A3m was devoid of bacterial colonization. Gram-Twort staining (Fig. 7F), colony growth (fig. S14, A and B), and shaking culture (fig. S14, C and D) after incubation of tissue homogenates demonstrated that no bacterial infection had occurred in hMPB, hMPB-A3m, or sCA. The PCL fibers within hMPB implanted in sheep were observed by SEM and showed no obvious fracturing, fragmentation, or morphological changes before and after AVG for 3 months (fig. S15A). However, assessment of PCL molecular weight in hMPB determined a decrease to 88.63% after 30 days of SI in sheep, which indicated a degradation rate of PCL similar to that of canine models after 30 days of SI (Fig. 5I). The molecular weight of PCL further decreased to 80.08% 3 months after AVG in sheep models (fig. S15B).

### Macrophage, microvasculature, and ECM analysis of hMPB after VI

In rat, canine, and sheep models, CD68^+^ macrophages (figs. S16, S17, and S18), α-SMA^+^ microvasculature (figs. S19, S20, and S21), and ECM [collagen, glycosaminoglycan (GAG), and elastin] (figs. S22, S23, and S24) showed uniform distribution throughout hMPB before VI. After VI, the overall number of detectable CD68^+^ macrophages (figs. S16, S17, and S18) and microvessels (figs. S19, S20, and S21) had significantly decreased in all models, and the number of microvessels at experimental end points was closer to that of the corresponding native arteries. After VI, ECM deposition in the neointima showed more circumferential alignment than before VI, akin to the tunica media of native artery (figs. S22, S23, and S24). Mature elastin content was assayed on the basis of detection of insoluble elastin content, in accordance with protocols described in previous studies (*30*, *31*). The mature elastin content had increased in comparison to pre-VI hMPB. The collagen and GAG content also demonstrated a remarkable increase with increasing VI time (figs. S22, S23, and S24). In canine models, ePFTE-V7m had large numbers of CD68^+^ macrophages (fig. S17) but no microvessels (fig. S20). In ePTFE grafts, ECM was found to only show disordered deposition in IH near the suture site without deposition in graft walls (fig. S23). All samples in canine and sheep models stained negative for calcification with von Kossa stain (figs. S23 and S24).

## DISCUSSION

Here, PB was generated by utilization of the FBR to subcutaneously embedded PS with differentially tuned structural characteristics (fiber winding angle and heat treatment–induced fiber fusion). We first established that hMPB performed the best as autologous vascular grafts in rAA replacement models (Figs. 1 and 2). In subsequent cCA replacement models (Figs. 4 and 5) and sheep AVG models (Figs. 6 and 7), hMPBs as autologous vascular grafts (i) exhibited 100% patency and observable compliance without thrombosis and IH; (ii) drove vascular regeneration (ECs coverage, contractile SMC layer formation, and ECM remodeling); (iii) integrated with host tissues (vascularization with minimal presence of macrophages); (iv) achieved functional regeneration after vascular grafting (physiological activity with ACh/SNP relaxation and KCl/AD contraction); and (v) were immediately available for cannulation and displayed resistance to repetitive puncture in AVG models.

Compared to autologous vessels, in vivo engineered biotubes have no branches and valves, which is preferential for clinical application; they are flexible and customizable with a high degree of precision and, thus, are suitable alternatives for a wide variety of vascular graft applications whereby numbers of autologous vessels are limited because of issues with quality, size, and prior use (*1*, *3*, *16*). However, poor mechanical properties of unsupported TB have often resulted in unsatisfactory results after implantation (*20*). Advancements have been made in improving the mechanical strength of TB. Reinforced biotubes were prepared with cage molds to improve wall thickness and mechanical strength, which lengthened the time of SI and required additional steps to remove cage mold components before use of the biotubes as vascular grafts (*32*). Another group used cyclic mechanical loading to stimulate ECM production and improve biotubes strength in situ, but the resultant biotubes showed signs of aneurysm after VI (*33*). More recently, the FBR was modulated using implants with modified surfaces, which resulted in increased TB wall thickness, collagen deposition, and myofibroblast activity (*22*). However, these biotubes still had mechanical issues, even after incorporation of PCL exterior wraps (*22*). Despite these attempts, the biotubes failed to achieve comprehensive and effective control of mechanical strength, which may be partly due to their only reliance on the production of ECM to bolster structural support.

PCL is a Food and Drug Administration–approved biodegradable polyester with excellent mechanical strength and biocompatibility (*29*, *34*). PCL artificial vascular grafts with different structures can be fabricated by regulating fiber deposition on the basis of electrospinning or wet-spinning techniques (*14*, *35*, *36*). However, it is difficult to accurately control fiber arrangement because of the inherent unpredictable nature of the electrified jet in solution electrospinning or the processing conditions of wet-spinning (*27*, *37*). Melt-spinning permits the precise control over fiber deposition and mechanical properties of tubular scaffolds (*27*, *29*). Therefore, we fabricated hMPS using melt-spinning and embedded them into subcutaneous pouches to exploit the FBR and form hMPB for use as autologous grafts. Our hMPB not only retained the advantages of the biotubes but also achieved comprehensive and effective control of mechanical strength (maintained the characteristics of tubular structures, easy to suture, resisted arterial pressure and kink formation, and appropriate compliance after VI).

The regulatory effects of the PS characteristics on the mechanical properties of the resultant reinforced biotubes can predominantly be attributed to the following two points: (i) the fusion between fibers and (ii) the fiber winding angle. The fiber skeleton without heat treatment had the appearance of loose fibers due to weak fusion between the fibers, which resulted in burst pressures lower than the clinical threshold (1600 mmHg) (Fig. 1I), and, thus, did not meet the requirements of vascular implants (*38*). Matsuda *et al.* (*39*) reported that contact fusion between fibers increased the mechanical strength of electrospinning vascular grafts. Accordingly, we improved fiber fusion in our PS by heat treatment (Fig. 1B, iii) to form hPS that had enhanced mechanical properties (Fig. 1, H and I). Heat treatment did not affect fiber diameter, winding angle, or pore size of PS (fig. S2). The fiber angle of hPS is causally related to axial and radial mechanical properties of hPB (Fig. 1, J and K). Higher radial modulus of vascular prostheses may be conducive to the induction of SMC proliferation and their transition from contractile to synthetic phenotypes, which was a suggested factor that increases the risk of calcification, IH, and vascular occlusion (*40*–*42*). One month after small-angle hPB was implanted in rAA replacement models, severe IH was observed (fig. S6), which may be reflective of the heightened radial modulus exhibited by the small-angle hPB (Fig. 1J). On the other hand, radial mechanical insufficiencies may also explain large-angle hPB development of aneurysms and vascular rupture within 1 month of VI in rat models (fig. S7). On the basis of the mechanical properties of hMPB (Fig. 1, J and K), we predicted that hMPB would form grafts with less risk of IH, aneurysm, and vascular rupture, thereby being favorable for vascular regeneration with increasing implantation time. The hMPB grafts, even after 3 months of implantation time in rats, remained patent without aneurysm or luminal stenosis (Fig. 2, A to F). The formed EC monolayers were arranged in parallel to blood flow direction (Fig. 2H), and the closely adhered SMC layers were aligned along the axial direction, which were consistent with native arterial tissue structure (Fig. 2I).

Early studies showed that nondegradable ePTFE grafts caused long-term inflammation in vivo, which can lead to calcification or IH, ultimately resulting in graft failure (*6*, *11*, *43*). We observed similar issues in this study with ePTFE grafts at 7 months after implantation in cCA. Numerous CD68^+^ macrophage cells were present in the graft walls (fig. S17), and almost no vascular regeneration was detected (Fig. 5, B to D). Following hMPB implantations in rat, canine, and sheep models, hMPS degradation did not result in exacerbated inflammation; on the contrary, the number of CD68^+^ macrophages significantly decreased over time (figs. S16, S17, and S18). Wang’s group (*44*) recently demonstrated that slow degradation of grafts inhibited acute inflammatory responses associated with degradation products from synthetic materials, which contributed to SMC regeneration, ECM remodeled to resemble native arteries, and robust mechanical properties. The complete in vivo degradation of PCL takes approximately 18 months. Slow degradation rates of polymers are considered beneficial in the avoidance of rapid mechanical loss during early remodeling stages of tissue regeneration (*34*, *45*). At 7 months after VI of hMPB (hMPB-V7m), the PCL fibers exhibited signs of fragmentation (Fig. 5H). The molecular weight of the PCL in hMPB-V7m grafts was found to have decreased by 27.95%, compared with that of the original construct, hMPS (Fig. 5I). In addition to the absence of aggressive accumulation of macrophages during PCL degradation (fig. S17), we observed significant increases in ECM deposition, including collagen, GAG, and mature elastin (fig. S23). Previously, it was shown that ECM deposition effectively compensated for loss of mechanical strength due to polymer degradation, and this helped avoid graft dilatation or rupture (*45*, *46*). In support of this, we found no signs of dilatation or rupture in hMPB-V7m (Fig. 4, B, E, and G). In native arteries, collagen contributes greatly to arterial tensile strength, whereas mature elastin provides elasticity and compliance. Thus, many efforts have been made to improve mature elastin deposition in vascular grafts to increase elasticity and compliance of grafts (*30*). Satisfactorily, we found that the mature elastin content of hMPB increased after VI in the three animal models. In the long-term cCA replacement model, the mature elastin of hMPB-V7m reached approximately 73% of native cCA. The deposition/remodeling of an organized mature elastin in tandem with PCL degradation contributed to this change; hMPB-V7m had reduced stiffness but enhanced elasticity compared to the pre-VI hMPB (Fig. 5J) and showed detectable compliance (Fig. 4E). More unexpectedly, the hMPB-V7m grafts achieved a J-shaped stress-strain curve similar to native arteries (Fig. 5J). These favorable changes in mechanical properties appeared to be more conducive to the maintenance of SMC contractile phenotypes, which was suggested to be essential in maintaining mechanical strength in vascular grafts (*29*, *40*).

In view of slow PCL degradation with beneficial vascular regeneration, we speculate that at the time of complete PCL degradation (suggested to occur approximately 18 months after VI), the hMPB grafts would have a stronger resemblance to native arteries and would be capable of resisting arterial pressure while preserving the compliance observed in hMPB-V7m. Future evaluation of hMPB grafts in large animal models beyond the point of complete PCL degradation is required to test our speculations. A potential caveat of using slowly degrading PCL may be associated with the inhibition of late-stage vascular remodeling and tissue growth potential (*14*), as suggested by our observations of restricted lumen diameter growth (Fig. 4E). Substitution of PCL with a rapidly degrading polymer of equivalent mechanical strength could serve as a potential solution. Previously, it was reported that the fast degradation of biomaterials facilitate macrophage-orchestrated tissue regeneration (*47*). Whether rapidly degrading polymer skeleton–reinforced biotubes can recapitulate the effective formation of an organized ECM and exhibit long-term resilience against arterial blood flow remains to be investigated.

The requirements of AVGs differ from arterial grafts. AVGs should be durable, should be resistant to puncture, and should facilitate rapid hemostasis (*48*). The commonly used ePTFE AVGs presented rupture points following their puncture (*49*–*51*). Satisfactorily, the precise structure of the hMPS enables the prepared hMPB to seal perforations quickly after being punctured with a 16G dialysis needle (Fig. 6B). Following ISO 7198, we performed 24 punctures in vitro, and the mechanical strength remained higher than sCA (Fig. 6C). Research groups, including Niklason and Tranquillo, used their TEVGs (6-mm diameter, ≈13-cm length) as AVGs in baboon models. During the 6-month implantation period, cannulation with a dialysis needle was performed thrice in Niklason’s study (*3*) and four times in Tranquillo’s study (*7*). These TEVGs were concluded to be candidate grafts with the most potential for use as AVGs due to the good patency, tissue regeneration, and resistance to repeated puncture (*3*, *7*). Furthermore, Niklason group’s TEBVs have yielded promising results in a phase 2 clinical trial as AVGs (*1*, *6*). In our study, hMPB grafts (5-mm diameter; 14- to 16-cm length) were implanted as AVGs into sheep (Fig. 6A), which have been described as having a coagulation system that is closer to human (*52*). Under careful guidance from the anesthesiologist, punctures with dialysis needles were made immediately after surgery and once a month thereafter. After each puncture, hemostasis was achieved within 5 min of applied pressure, suggesting good puncture closure (Fig. 6D and figs. S11 and S12). We systematically tested our AVGs and performed punctures (a total of four times), once per month. Adequately, no dilatation, rupture, or thrombus formation was observed in hMPB at 3 months after AVG grafting (Fig. 6, G, I, and J).

In sheep AVG models, we simulated remote patient treatment, as would be performed by an on-site general surgeon. We delivered hMPS to the institution that housed the sheep. A general surgeon easily completed the SI of the hMPS, according to our predesigned surgical program, which did not require sophisticated equipment or technology to complete (a procedure similar to SI of contraceptive devices or glucose sensors). After 1 month, the formed hMPB was auto-implanted as AVG by a vascular surgeon. This is a timely process, which would be equivalent to cases wherein patients are referred from a local hospital after SI, to an advanced general hospital for the AVG grafting 1 month later. The time and procedures are also applicable to using hMPB as arterial replacements/bypass. Our AVGs were available for puncture and cannulation immediately after their grafting (Fig. 6, D and E, and fig. S11), providing a prompt access for hemodialysis, which is a stark contrast to the several weeks of maturation time required by ePTFE grafts before cannulation (*7*). In addition, hMPBs were prepared according to the predesigned tunnel (Fig. 6A), only a small part of the adjacent arteries and vein was separated from subcutis to perform the AVG, and this is comparable to tissue trauma accrued by AVG using ePTFE grafts (*53*). Commercially available ePTFE grafts are known to provoke inflammatory reactions and have issues with host-graft integration that are susceptible to bacterial infections (*2*, *7*, *43*, *54*). Our results indicated that no bacterial infection occurred in hMPB after 3 months of AVG grafting (Fig. 7F and fig. S14), which is more likely to be due to the agreeable ECM remodeling and host integration of the implanted grafts (*6*, *48*). On the basis of the above analyses, we believe that our hMPB is a promising candidate for clinical application as AVG.

Although this study showed beneficial results, it had several limitations. For example, hMPB evaluation was performed in a limited number of large animal models (*n* = 3 in cCA replacement models and sheep AVG models). In addition, to reduce the number of anesthetic events, puncture tests were restricted to once per month in the sheep AVG model. Our in vivo/in vitro puncture experiments make us confident that our hMPBs would meet the clinical need for AVG, but it remains to be seen whether the hMPB can tolerate the triweekly cannulation required for hemodialysis. Furthermore, although hMPBs continued to demonstrate patency after AVG grafting, the AVG experiments were prematurely terminated at 3 months because of the unfortunate timing of the coronavirus disease 2019 (COVID-19) epidemic, and rapid implantation of epidemic prevention policies restricted travel and access and prevented the required personnel from attending the sheep for longer-term assessment of AVG. Thus, longer-term experiments are still required to systematically evaluate the performance of hMPB as AVGs.

Inspired by the architectural engineering method of embedding steel construct supports into concrete, we successfully fabricated hMPB using the subcutis of different animals. The hMPB could be custom-made to meet personalized implantation. In rAA replacement models, cCA replacement models, and sheep AVG models, our hMPB demonstrated satisfactory patency without thrombosis, IH, and dilatation while exhibiting impressive vascular regeneration and host integration. hMPBs had qualities ideal for use as alternatives to autologous vessels and hold promise for clinical transformation.

## MATERIALS AND METHODS

### Experimental design

This study aimed to provide a method to generate PB with good mechanical properties and vascular regeneration for arterial replacement and arteriovenous grafting. First, we fabricated PSs with different structures and embedded them into rat dorsal subcutaneous pouches for 30 days to form different types of PB. We screened out the optimal PB (hMPB) by evaluating the mechanical properties (suture retention strength, burst pressure, radial, and axial mechanical properties) and regenerative properties in a rat autologous abdominal aorta implantation model (*n* = 5). The hMPBs as autogenous grafts were further investigated in canine models of peripheral arterial replacement (*n* = 3) and sheep models of AVG for hemodialysis (*n* = 3). For canine and sheep, surgery was performed by senior surgeons; CDU, angiography, CT, and MRI were performed and interpreted by medical imaging experts blinded to the treatment.

### PS fabrication and characterization

PS was prepared by melt-spinning. Briefly, PCL pellets (*M*_n_ = 70,000 to 90,000 Da; Sigma-Aldrich) were added into a 20-ml stainless steel syringe with a stainless steel 16G flattened needle tip, which was placed above the collector at a distance of 1 cm. The PCL pellets were heated at 150°C for 1 hour to achieve a homogenous PCL melt. The feed rate of PCL melt was controlled using a syringe pump (Cole-Parmer US). The collector (silicone tubes with steel bar central insert) was connected to a stepper motor to provide rotation, and this was mounted on top of a linear slide to provide lateral roundtrip translation. Different combinations of rotational and lateral translation speed of the collectors and PCL feed rates were used to adjust fiber winding angle (the angle between fibers of two adjacent layers) (table S2). These fabrication parameters were used to prepare desired PS with fiber diameter ≈ 60 μm and different fiber winding angles (small angle ≈ 30°, medium angle ≈ 50°, and large angle ≈ 110°). PSs were immersed in a 56°C water bath for 3 s to reinforce fusion bonding among fibers (heat treatment) before rapid immersion in ice-water mixture (0°C). nPS or hPS was dried under nitrogen. The steel bar was replaced with silicone rods of equivalent diameter, and the PS was tightened with 3-0 sutures at both ends. Templates consisting of PS, silicone tubes, and silicone rods were sterilized with ethylene oxide before use in experiments.

### The characterization of PS with or without heat treatment characterization

PSs, including nPS and hPS, were mounted on aluminum stubs and sputter-coated with gold. SEM (Phenom Pro, Phenom-World BV, Eindhoven, the Netherlands) at an accelerating voltage of 15 kV was used to observe the fiber structure of outer surfaces of the PS. On the basis of the SEM images of the outer surfaces of PS, the fiber diameter, pore diameter, and fiber winding angle were analyzed using Image-Pro Plus 6.0 software. At least five fibers and the angle between the fibers of two adjacent layers per image, three images per sample, and five samples per group were included to calculate the fiber diameter and fiber winding angle, respectively. The pore size was measured according to previously described procedures (*55*). Briefly, the area of the pores between fibers of the topmost graft layer was calculated and normalized to circularized areas. Pore diameter was obtained from the circle area. At least three pores per image, five images per sample, and five samples per group were used to calculate the average pore diameter. The change of bonding among fibers before and after heat treatment was also observed by SEM at high magnification.

### Animal approval and anesthesia protocols

Experiments on rats (male, 280 to 320 g, 8 to 10 weeks), rabbits (male, 2 to 3 kg, 3 to 4 months), and beagle dogs (male, 9.5 to 10.5 kg, 8 to 12 months) were approved by the Animal Care and Use Committee of Nankai University, and sheep (male, 30 to 35 kg, 5 to 6 months) experiments were approved by the Animal Care and Use Committee of Tianjin Union Medical Center. All animal housing and experiments were performed under humane care, in accordance with the NIH *Guide for the Care and Use of Laboratory Animals*.

Rats were anesthetized through intraperitoneal administration of sodium pentobarbital (60 mg/kg) (*56*). Rabbits were anesthetized by intravenous injection of sodium pentobarbital solution (30 mg/kg) (*57*). In dog experiments, anesthesia was induced by intramuscular injection of xylazine (1.5 mg/kg). Sheep were anesthetized with intramuscular injection of xylazine (2 mg/kg) and midazolam (0.05 mg/kg). After endotracheal intubation, dogs and sheep were maintained under general anesthesia by inhalation of isoflurane (1 to 4%) (*32*). Anesthesia was induced in animals before SI and VI/AVG.

### Fabrication of PS-reinforced biotubes

Templates with inner diameters of 2, 3, and 5 mm were implanted subcutaneously in the dorsum of rats (nPS and hPS), rabbits (hMPS), dogs (hMPS), and sheep (hMPS), respectively. A small incision was made, and a template of appropriate length was inserted into the subcutaneous pouch. The skin was closed intracutaneously. Thirty days after SI, templates and the surrounding tissue capsules were gently removed from the subcutaneous tissue. After harvest, the ends of the templates were blunted. Then, PBs, including nPB and hPB, were obtained by removing silicone tubes and silicone rods from the tissue capsule. Specialized shape templates were embedded into subcutaneous pouches of rabbits to form hMPB. Silicone rods (outer diameter 2 mm) were used to fabricate TB in rats. The morphological characterization of nPS, hPS, nPB, hPB, TB, and rAA was observed by stereomicroscopy (S8AP0, Leica). Body weight and temperature of all rats were recorded before and after SI.

### Rat body weight and temperature monitor

After nPS and hPS were implanted subcutaneously, the body weight and temperature of all the rats were recorded at 0, 1, 4, 7, 14, and 28 days. Body weight was measured at the beginning of the dark period each day. Rectal temperature of each animal was monitored by a digital thermometer. The normal rat (without any surgery) and sham rat (surgery alone) as control were tested using the same protocol.

### Measurement of wall thickness and inner diameter of nPS, nPB, hPS, and hPB

The outer and luminal perimeters of the nPS, hPS, nPB, and hPB were manually measured from stereoscope images of the cross section with Image-Pro Plus 6.0 software to determine the luminal diameter and wall thickness of the nPS, nPB, hPS, and hPB. At least five samples per group were used to calculate the average wall thickness and inner diameter.

### Mechanical characterization

Mechanical tests were performed immediately after obtaining nPB/hPB from animal models. The mechanical testing of graft samples obtained from rats included suture retention strength, burst pressure, and longitudinal/radial mechanical properties. For samples obtained from sheep, strength after repeated puncture and suture retention strength were tested in accordance with ISO 7198:2016. These tests were performed in quintuplicate. The detailed procedure of mechanical tests is available in the Supplementary Materials.

### Vascular implantation

Each hPB with three different winding angles (2-mm diameter, 1.1-cm length) as autogenous grafts was end-to-end implanted into rAA using interrupted 9-0 monofilament nylon sutures. TBs without PS support were used as controls (2-mm diameter, 1.1-cm length) and were also auto-implanted into rAA using the same protocol. No anticoagulants or antiplatelet agents were administered to the rats post-operatively. hMPB showed optimal vascular regeneration in rats at 1 month and further evaluated in larger animal models.

Canines are suitable large animal models for long-term evaluation of small-diameter vascular grafts because canine peripheral arteries fall within the diameter range of 3 to 5 mm, and the animals themselves are tolerant to prolonged anesthesia (*52*). Therefore, hMPBs (3-mm diameter, 3.5-cm length) were auto-implanted into cCA in an end-to-end fashion using 7-0 prolene sutures via continuous suture to evaluate long-term in vivo patency and regenerative ability of hMPB. Control ePTFE grafts (3-mm diameter, 3.5-cm length, GORE-TEX, WL Gore & Associates) were implanted into cCA using the same protocol. Canines received dual antiplatelet therapy (162.5 mg of aspirin/37.5 mg of clopidogrel), daily preoperatively until the end of the study (*3*). Experimental end points of canine models were set at 7 months.

Sheep are frequently used large animal models for establishing AVG due to the possession of similar immunological systems and inflammatory elicitations to humans. In addition, sheep have accessible and long neck vessels that are suitable for the evaluation of longer-length vascular grafts (*58*). To generate AVG models, a subcutaneous tunnel (approximately 17- to 19-cm length) was prepared in one side of the neck of each sheep. The ends of the subcutaneous tunnel were predesigned to connect to carotid artery and jugular vein. One template was implanted per subcutaneous tunnel. Thirty days after SI, the two ends of the formed tissue capsule surrounding the hMPSs were separated from the surrounding subcutaneous tissue, and the midportion of the tissue capsule remained intact and integrated with subcutaneous tissue. hMPBs were obtained according to the predesigned position by removing silicone tubes and silicone rods from the tissue capsules. hMPBs (5-mm diameter, 14- to 16-cm length) were auto-implanted as end-to-side AVG between the carotid artery and jugular vein using a continuous 7-0 prolene suture. Sheep received low–molecular weight heparin [Qilu, 15,000 IU/head, subcutaneously, once daily (QD)] starting the same day as AVG, and clopidogrel (300 mg per head for 7 days before AVG, then 150 mg per head every day thereafter, orally, QD) (*59*). Experimental end points of sheep models were at 3 months due to laboratory and animal house restraints put in place as part of COVID-19 epidemic prevention policies. All animals received heparin (100 UI/kg) at the time of VI/AVG.

### Color Doppler ultrasound

In rat, canine, and sheep models, CDU (rat: Vevo 2100 System Visualsonics, Canada; canine and sheep: Mindary, M9, China) was used to monitor patency, lumen diameter, and flow velocity of hMPB at predetermined time points.

### Angiography, CT, and MRI

At 7 months, angiographies of canine hMPB and ePFTE grafts were acquired using a GE C-arm (OEC 9900 Elite) after injection of contrast media (iopromide; via percutaneous femoral artery). In canine models, 3D CT angiography was performed to detect in vivo 3D appearance of hMPB and ePFTE grafts at 7 months after VI. Images were obtained using a 256-slice helical CT scanner (GE Revolution CT) with injected contrast media (iohexol; via cephalic vein). 3D reconstructions were performed at a dedicated workstation. At 7 months, MRI of canine hMPB and ePFTE grafts was performed using a 3-T Magnetom Prisma system (Siemens Healthcare) with a standard 64-channel head coil.

### In vivo compliance

Implanted hMPB compliance in rat and canine models was calculated using the following equation

$$\%\;\text{compliance}\;\text{per}\;100\;\text{mmHg}=\frac{(Rp_{1}-Rp_{2})/Rp_{1}}{p_{2}-p_{1}}\times 10^{4}$$ (1)

where *p*_1_ is low pressure value (mmHg), *p*_2_ is high pressure value (mmHg), and *Rp*_1_ and *Rp*_2_ are the inner diameter of hMPB at the respective pressure. Procedures for blood pressure measurements and inner diameter calculations were performed according to previous reports (*14*, *34*). Briefly, the rat blood pressure was measured with a noninvasive tail cuff system (Softron, BP-2010 Series, Blood pressure meter, Softron Biotechnology, Beijing, China). The canine blood pressure was measured by noninvasive blood pressure monitoring using an electrocardiogram monitor (BeneView T5, Mindray, China). The inner diameters of the implanted hMPB at low and high pressures were measured from the recorded video frames taken by CDU.

### Cannulation of AVG

In sheep AVG models, hMPB was cannulated with a 16G dialysis needle immediately after surgery and at 1, 2, and 3 months. Needle placement was verified with blood flow and flush patency. After removal of the needle, the cannulation site was compressed until hemostasis was reached. The time to hemostasis was recorded.

### hMPB harvest and sample allocation

In rat models, hMPBs before and after VI were harvested and cut into two halves. One-half was then cut into two ring samples. One ring sample was snap-frozen in optimal cutting temperature (OCT) compound (Tissue Tek) for cross-sectioning. The other ring sample was used to test vascular function and ECM quantification. The remaining half was longitudinally cut into two pieces. One piece was observed by stereomicroscopy and then snap-frozen in OCT for longitudinal sectioning. The other pieces were prepared for SEM analysis. The frozen samples were cut to a slice thickness of 6 μm for histological analysis.

In the canine model, hMPB, hMPB-V7m, and ePTFE-V7m were harvested for histological analysis, vascular functional testing, ECM quantification, SEM analysis, PCL degradation, mechanical testing, and protein extraction for WB. In sheep models, hMPB and hMPB-A3m were harvested, halved longitudinally, and flattened to determine the presence of thrombus formation. Then, hMPB-A3m halves were cut into samples for histological analysis, ECM quantification, SEM analysis, and infection detection. For histological analyses, the samples from canine and sheep models were fixed in 10% neutral buffered formalin, embedded in paraffin, and sliced to 5-μm-thick sections.

### Histological analyses

Tissue sections from rat, canine, and sheep were stained with H&E using standard protocols. Von Kossa and Gram-Twort staining of sections were used to evaluate calcification (canine and sheep explants) and bacterial infection (sheep explants), respectively.

For ECM remodeling and quantification, tissue sections were first stained with Masson’s Trichrome, Safranin O, or Verhoeff van Gieson (VVG) using standard protocols. The collagen, GAG, and mature elastin (insoluble elastin) content of samples from rats, canines, and sheep were detected using Sircol Collagen Assay Kit (S111, Biocolor), Blyscan GAG Assay Kit (B1000, Biocolor), and Fastin Elastin Assay Kit (F2000, Biocolor) (*30*, *31*), according to the manufacturer’s instructions.

Frozen sections were fixed in cold acetone for 10 min, air-dried, and washed with phosphate-buffered saline (PBS). Paraffin sections were deparaffinized and rehydrated, and then antigen retrieval was performed using heat-mediated citrate buffer (pH 6). To stain cell surface antigens for IF, slides were blocked using 5% normal goat serum for 45 min at room temperature, and then incubated with anti-CD31 antibody (rat explants: Abcam, ab64543, 1:100 dilution), anti-eNOS antibody (rat explants: Thermo Fisher Scientific, PA5-16887, 1:100 dilution), or anti-CD68 antibody (rat explants: Abcam, ab125212, 1:100 dilution; canine explants: Thermo Fisher Scientific, MA1-81381, 1:200 dilution; and sheep explants: Thermo Fisher Scientific, MA1-81381, 1:200 dilution) in PBS for 12 hours at 4°C. To stain intracellular antigens, slides were pretreated with 0.1% Triton X-100–PBS to permeate the membrane before blocking and subsequent incubation with anti–α-SMA antibody (rat explants: Abcam, ab7817, 1:100 dilution; canine explants: Abcam, ab7817, 1:200 dilution; sheep explants: Abcam, ab7817, 1:200 dilution), anti-MYH antibody (rat explants: Santa Cruz Biotechnology, sc-6956, 1:100 dilution), anti-vWF antibody (canine explants: Abcam, ab6994, 1:100 dilution; sheep explants: Abcam, ab6994, 1:100 dilution), and anti-CNN antibody (sheep explants: Abcam, ab46794, 1:100 dilution) in PBS for 12 hours at 4°C. After incubation with primary antibodies, the sections were rinsed with PBS (five washes) and then incubated with Alexa Fluor 488–conjugated goat anti-mouse immunoglobulin G (IgG) (Invitrogen, A-11029, 1:200) and/or Alexa Fluor 594–conjugated goat anti-rabbit IgG (Invitrogen, A-11037, 1:200) antibodies in PBS for 2 hours at room temperature. After washing with PBS (five washes), the sections were stained with a 4′,6-diamidino-2-phenylindole–containing mounting solution. Sections without primary antibody incubation were used as negative controls for background staining.

For immunohistochemistry (IHC) staining, paraffin-embedded tissue sections were deparaffinized and rehydrated. Then, the sections were incubated with 3% H_2_O_2_ for 10 min and washed three times in PBS. Sections were permeated with 0.5% Triton X-100–PBS for 10 min and then washed three times in PBS. Sections were then blocked with 5% normal goat serum for 45 min at 4°C and then incubated with mouse anti-MYH (canine explants: Abcam, ab212657, 1:100 dilution) for 12 hours at 4°C. After washing five times in PBS, slides were then incubated with horseradish peroxidase–labeled goat anti-mouse IgG (H + L) antibody (Bioworld, bs12478, 1:200) for 2 hours in darkness. Antibody binding was visualized by incubation with a 3,3′-diaminobenzidine chromogen kit (ZSGB-Bio, China) and then counterstained with hematoxylin for 5 min.

For IF staining, slides were observed under a fluorescence microscope (Zeiss Axio Imager Z1, Germany), and images were acquired with a digital camera (AxioCam MRm, Germany). For H&E, Masson’s trichrome, Safranin O, VVG, von Kossa, Gram-Twort, and IHC-stained sections, slides were observed under an upright microscope (Leica DM3000, Germany), and images were acquired with a digital camera (Leica DFC450, Germany).

All histology was interpreted by a blinded cardiovascular pathologist. The detailed information of the statistical analyses used to validate changes in neointima thickness, eNOS^+^ ECs coverage, contractile SMCs thickness, the number of CD68^+^ macrophage, and microvasculature is available in the Supplementary Materials.

### Vascular function

After hMPBs were implanted into rats’ abdominal artery for 3 months and cCA for 7 months, the physiological functions of regenerated and native arteries were assessed by aortic ring bioassay using a PowerLab/870 eight-channel 100-kHz A/D converter (AD Instruments, Sydney, Australia) according to previously described procedures (*35*). The detailed procedure is available in the Supplementary Materials. Results were obtained from three individual rings of the explants.

### Western blot

WB was performed to semiquantify α-SMA and MYH protein expression of hMPBs before and after implantation into cCA for 7 months as the protocol of our previous report (*55*). ePTFE grafts after implantation into cCA for 7 months and natural cCA as control were also tested using the same protocol. The detailed procedure is available in the Supplementary Materials.

### Polymer degradation analysis

To evaluate PCL degradation, the hMPS and hMPB before and after VI in canine and sheep models were examined by GPC according to our previously reported protocol (*14*). In brief, the samples were dissolved in tetrahydrofuran for 72 hours under agitation and filtered with a 0.22-μm filter membrane (Nylon66, Ameritech). The molecular weight of the extracted polymer was analyzed by GPC (Waters, USA). The PCL fiber’s morphological characteristic of hMPB before and after VI was observed by SEM.

### Explant SEM observation

The samples were fixed in 2.5% (v/v) glutaraldehyde for 12 hours and dehydrated in ascending series of ethanol. Then, the samples were mounted onto aluminum stubs, sputter-coated with gold, and observed by SEM (Phenom Pro, Phenom-World BV, Eindhoven, the Netherlands).

### Detection of bacterial infection in hMPB AVG explants

Infection detection of hMPB as AVG in the sheep model was carried out according to a previous study (*60*). The detailed procedure is available in the Supplementary Materials.

### Statistical analysis

GraphPad Prism v5.0 (GraphPad Software) was used for statistical analysis. Single comparisons between two independent datasets were carried out using an unpaired Student’s *t* test. Multiple comparisons across one variable were performed using a one-way analysis of variance (ANOVA), and multiple comparisons across two variables were performed using a two-way ANOVA. All ANOVA analyses were followed by Tukey’s post hoc analyses. Data are expressed as the means ± SE. In two-way ANOVA, “^#^” denotes comparisons between different groups and “^+^” indicates comparisons within the same group. For all tests, *P* < 0.05 was considered significant. Figure legends indicate the statistical tests used for data analysis.
