# Supplementary material for: Mechanically reinforced biotubes for arterial replacement and arteriovenous grafting inspired by architectural engineering
Source: Sci Adv. 2022 Mar 16;8(11):eabl3888. doi: 10.1126/sciadv.abl3888 (PMC8926343; doi:10.1126/sciadv.abl3888)
Supplement: Supplementary file 1 — Supplementary Materials and Methods Figs. S1 to S24 Tables S1 and S2 [file sciadv.abl3888_sm.pdf]

Science Advances
AAAS

Supplementary Materials for

Mechanically reinforced biotubes for arterial replacement and arteriovenous grafting inspired by architectural engineering

Dengke Zhi, Quhan Cheng, Adam C. Midgley, Qiuying Zhang, Tingting Wei, Yi Li, Ting Wang, Tengzhi Ma, Muhammad Rafique, Shuang Xia, Yuejuan Cao, Yangchun Li, Jing Li, Yongzhe Che, Meifeng Zhu*, Kai Wang*, Deling Kong*

*Corresponding author. Email: kongdeling@nankai.edu.cn (D.K.); 013053@nankai.edu.cn (K.W.); zhumeifeng2013@163.com (M.Z.)

Published 16 March 2022, *Sci. Adv.* **8**, eabl3888 (2022)
DOI: 10.1126/sciadv.abl3888

The PDF file includes:

Supplementary Materials and Methods
Figs. S1 to S24
Tables S1 and S2

Other Supplementary Material for this manuscript includes the following:

Movies S1 and S2

Supplementary Materials and Methods

Mechanical characterization

Mechanical tests were performed immediately after obtaining non-heat-treated PCL fiber skeleton (nPS)-reinforced biotubes (nPBs) and heat-treated PCL fiber skeleton (hPS)-reinforced biotubes (hPBs) from animal models.

The mechanical tests included evaluation of suture retention strength, burst pressure, longitudinal/radial mechanical properties. For suture retention strength, 6-0 prolene sutures were stitched and looped 2 mm from the cut ends of nPB/hPB from rats. Suture loops were clamped into an upper grip and nPB/hPB were immobilized in the lower grip. The samples were pulled at a crosshead speed of 8 mm/min. The force needed to displace the suture from nPB/hPB was recorded. For burst pressure, nPB/hPB obtained from rats were tested according to a previous report (*55*). In brief, immediately after nPB/hPB were harvested, burst pressure was measured by filling a nPB/hPB of 3 cm in length with soft paraffin (Vaseline), clamping one end and hermetically sealing the other end to a vascular catheter. After the sample was incubated at 37°C for 30 min, the Vaseline was filled at a rate of 0.1 mL/min. The filling pressure was recorded until the nPB/hPB wall burst. Longitudinal and radial mechanical properties of hPB obtained from rats were tested using a tensile-testing machine with a load capacity of 100 N (Instron-33) according to a previous report (*55*). For longitudinal mechanical properties, the hPB obtained from rats (3.0-cm-length; 1.0-cm-gauge length) were pulled longitudinally at a strain rate of 20 mm/min until rupture. For radial mechanical properties, hPB obtained from rats with 0.3-cm-length were fixed on two steel

rings which were clamped by machine chucks, and then pulled radially at a rate of 10 mm/min until rupture. Tensile strength and ultimate elongation at break were measured. The tensile strength was set as the peak stress of the stress-strain curve. Young's modulus was calculated from the initial linear region (up to 5% strain) of the stress-strain curve.

For samples obtained from sheep, strength after repeated puncture and suture retention strength was tested in accordance with ISO 7198:2016. Briefly, heat-treated medium-fiber-angle PB (hMPB) was punctured 0, 8, 16 and 24 times with a 16G dialysis needle in accordance with ISO 7198:2016 A.5.2.7 "*strength after repeated puncture*". Then, radial tensile strength was tested as above. Suture retention strength, including straight-across and oblique procedures, was conducted in accordance with ISO7198:2016 A.5.7 "*suture retention strength*". The straight-across procedure to assess suture retention followed the same method used to assess the samples obtained from rats. For the oblique procedure, hMPB was cut at an angle of 45 ° to the axis, and at the point 2 mm from the cut surface, a 6-0 prolene suture was pushed through the hMPB piece. The hMPB and the suture wire were fixed on a tensile testing machine and pulled with the same parameters as the straight-across procedure at three points of the hMPB piece – toe, 90 °, and heel positions. Mechanical tests were performed in quintuplicate.

Vascular function

After hMPBs were implanted into rat abdominal arteries for 3 months and canine carotid arteries for 7 months, the physiological function of regenerated and native arteries was

assessed by aortic ring bioassay using a PowerLab/870 Eight-channel 100kHz A/D converter (AD Instruments, Sydney, Australia). Sample rings of 3 mm length were submerged in Krebs buffer (composition in mM: NaCl, 118.4; KCl, 4.7; $CaCl_2$, 2.5; $MgSO_4$, 1.2; KH_2PO_4, 1.2; $NaHCO_3$, 25; dextrose, 11.1; Na_2Ca EDTA, 0.029; pH 7.4) at 37℃ and gassed with carbogenic mixture (95% O_2 and 5% CO_2). All preparations were stabilized under a resting tension of 2 g for 1 h with the buffer changed every 15 min. The presence of functional smooth muscle cells was indicated by the contractile responses induced by addition of potassium chloride (KCl) (60 mM). The function of the neo-endothelium was confirmed by the relaxation induced by acetylcholine (ACh) (10 μM), in pre-constricted segments by adrenaline (AD) (1 μM), and vascular smooth muscle function was evaluated by vascular relaxation in response to sodium nitroprusside (SNP) (1×10^{-7} mol/L). Isometric forces were recorded with force transducers connected to a PowerLab/870 Eight-channel 100 kHz A/D converter (AD Instruments). The physiological function of as prepared hMPBs in subcutis of rats and canine models, and expanded polytetrafluoroethylene (ePTFE) grafts after implantation into canine carotid artery for 7 months was measured using the same protocol.

Western blot

Western blot was performed to semi-quantify α-smooth muscle actin (α-SMA) and smooth muscle myosin heavy chain (MYH) protein expression of hMPBs before and after implantation into canine carotid artery for 7 months. In brief, total protein was extracted from samples using a Tissue Protein Extraction kit (Thermo Fisher Scientific, USA) containing

protease inhibitors. The lysates were centrifuged at 12,000 rpm for 10 min at 4 ℃ and the supernatants were transferred to fresh 1.5 mL tubes. Protein concentrations were determined by BCA assay (Solarbio, China). After boiling for 5 min with SDS-PAGE loading buffer, the protein samples were separated by electrophoresis using 4–12% SDS-PAGE gels. The separated proteins were transferred to a PVDF membrane (Merck Millipore, USA) and incubated with the appropriate primary antibodies overnight at 4 ℃. Primary antibodies were mouse anti-α-SMA (Abcam, ab7817, 1:1000), rabbit anti-MYH (Abcam, ab212657, 1:2000), and mouse anti-β-actin (Abcam, ab6276, 1:5000). The membranes were washed five times with PBS-Tween (PBST) before incubation with the appropriate horseradish peroxidase (HRP)-conjugated secondary antibody (HRP-goat anti-mouse IgG (H+L) antibody, Bioworld, bs12478, 1:5000; or HRP-goat anti-rabbit IgG (H+L) antibody, Bioworld, bs13278, 1:5000) for 2 h at room temperature. Following a further six washes with PBST, bound antibodies were detected using the Immobilon Western HRP detection reagents (Merck Millipore) and a Tanon-5500 Chemiluminescent Imaging System (Tanon, China). The intensities of all bands were quantified using Image-Pro Plus 6.0 software. ePTFE grafts implanted into canine carotid artery (cCA) for 7 months and native cCA tissue were used as controls and assessed using the same protocol.

Quantitative analysis of neointima thickness

Neointima thickness was measured using Image-Pro Plus 6.0 software based on hematoxylin and eosin (H&E) stained sections. For rats, three high-magnification images per sample and

five samples per group were included to obtain the statistical results. For canine, three whole cross-sections images per sample and three samples per group were included to obtain the statistical results. For sheep, four low-magnification images per section, three sections per sample and three samples per group were included to obtain the statistical results.

Quantitative analysis of endothelial cells (ECs) coverage rate

For rats, the ECs coverage rate was quantified by adding the length of endothelial nitric oxide synthase (eNOS)-positive monolayer and dividing this sum by the length of the longitudinal section of the hMPBs. Three sections per sample and five samples per group were included to obtain the statistical results.

Quantitative analysis of contractile SMCs thickness

For rats, the contractile smooth muscle cells (SMCs) thickness was analysed by immunofluorescence (IF) staining of the cross sections of the explanted hMPBs using anti-MYH antibody. Four high-magnification images per section, three sections per sample and five samples per group were included to obtain the statistical results. For canine, the contractile SMCs thickness was analysed by immunohistochemistry（IHC) staining of the cross sections of the explanted hMPBs using anti-MYH antibody. Four high-magnification images per sections, three sections per sample and three samples per group were included to obtain the statistical results. For sheep, the contractile SMCs thickness was analysed by IF staining of the cross sections of the explanted hMPBs using calponin (CNN) antibody. Four

low-magnification images per sections, three sections per sample and three samples per group were included to obtain statistical results.

Quantitative analysis of $CD68^+$ macrophage number

After cross-sections of samples were stained with anti-CD68 antibody, all $CD68^+$ cells within low-magnification images were counted to assess the inflammatory state of tissue samples. For rat tissue sections, four images per section, three sections per sample and five samples per group were included to obtain the statistical results. For canine and sheep, four images per section, three sections per sample and three samples per group were included to obtain the statistical results.

Quantitative analysis of micro-vessels formation

After cross-sections of samples were stained with anti-α-SMA, all α-SMA^+ microvasculature within low-magnification images were counted to assess micro-vessels formation. For rat tissue sections, four images per section, three sections per sample and five samples per group were included to obtain the statistical results. For canine and sheep, four images per section, three sections per sample and three samples per group were included to obtain the statistical results.

Detection of bacterial infection in hMPB arteriovenous grafts (AVG) in sheep models

The corresponding tissue samples were weighed and homogenized using a tissue homogenizer (Heidolph Diax 900) in 10 mL buffered peptone water/g sample. A total volume of 100 μL of homogenate was plated and spread onto standard agar plates. Colony growth was observed after incubation at 37℃ overnight. In addition, 100 μL of the homogenate was added to Luria-Bertani (LB) liquid medium, incubated at 37℃ with an orbital shaker speed of 150 rpm for 8 h. Then, 150 μL of bacterial medium from each sample was transferred to a 96-well plate. The OD value at 600 nm was measured with a BioTek Microplate Reader. For the above analysis, pure LB without sample homogenate and LB inoculated with *S. aureus* (10^6 colony forming units (CFUs)/mL) were used as negative and positive controls, respectively.

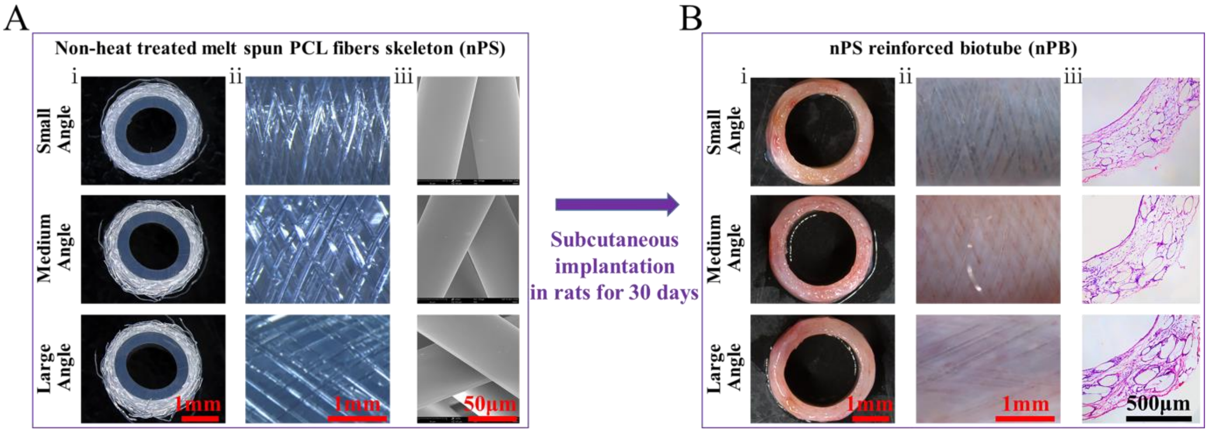

fig. S1. The appearance of nPS and nPB. (A) The morphological characterization of nPS. Stereoscope images of cross-section (i) and outer surface (ii) of nPS. High-magnification outer surface scanning electron microscopy (SEM) images showing the weak fusion at the fibers crossover points (iii). (B) The morphological characterization of nPB. Stereoscope images of cross-section (i) and outer surface (ii) of nPB. H&E-stained cross-section of nPB showing the complete tissue capsule formation in all three kinds of nPS (iii).

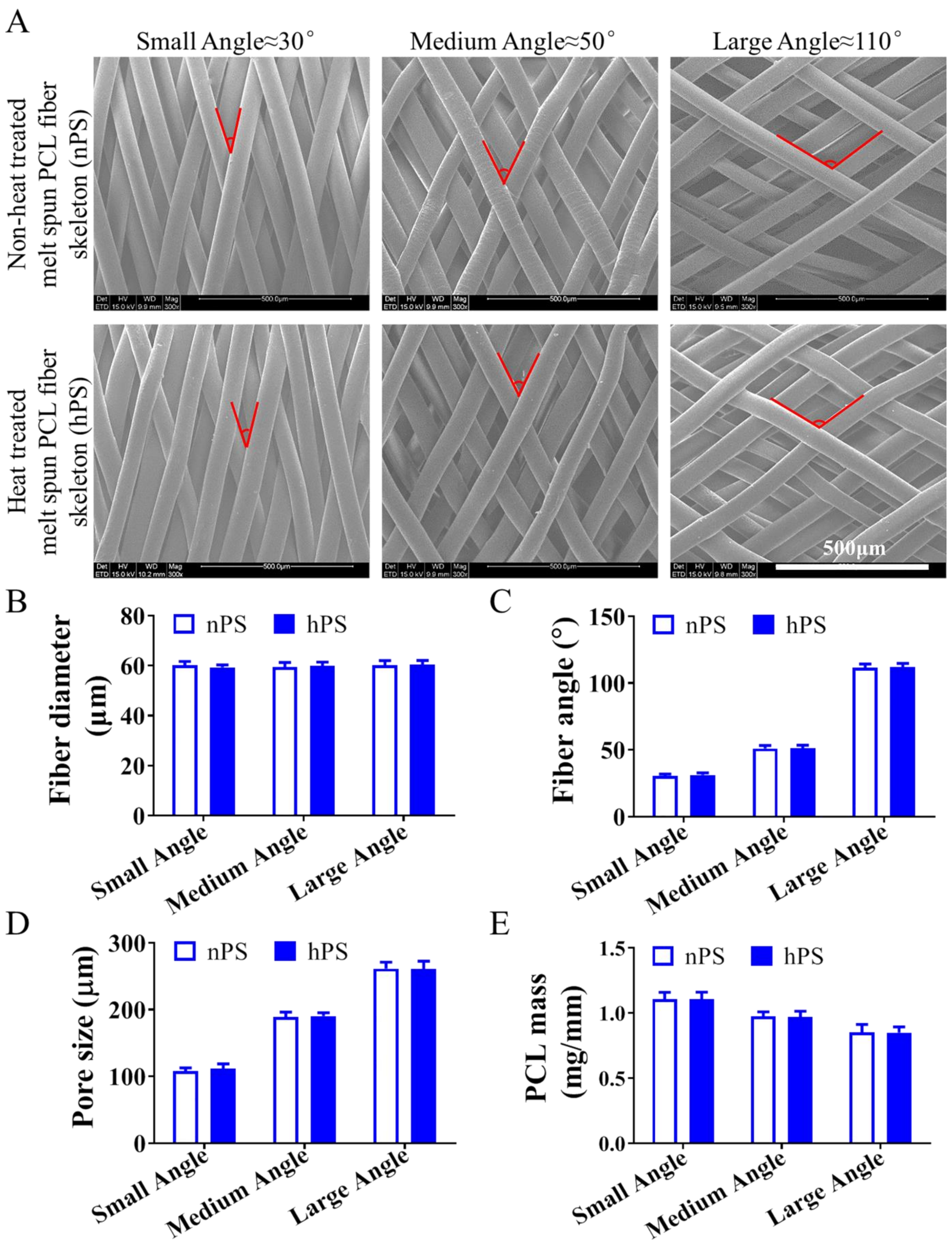

fig. S2. The fiber morphological characterization and PCL mass detection of nPS and hPS with three different fibers winding angles. (A) SEM images of the outer surface of nPS and hPS with three different fibers winding angle. Red lines represent the fibers winding

angle. (B to D) Quantitative analysis of fibers diameter (B), fibers winding angle (C) and pore size (D) of nPS and hPS with three different fibers winding angles based on the SEM images (n=5). (E) Quantitative analysis of PCL mass (mg/mm length) of nPS and hPS with the three different fiber winding angles (n=5).

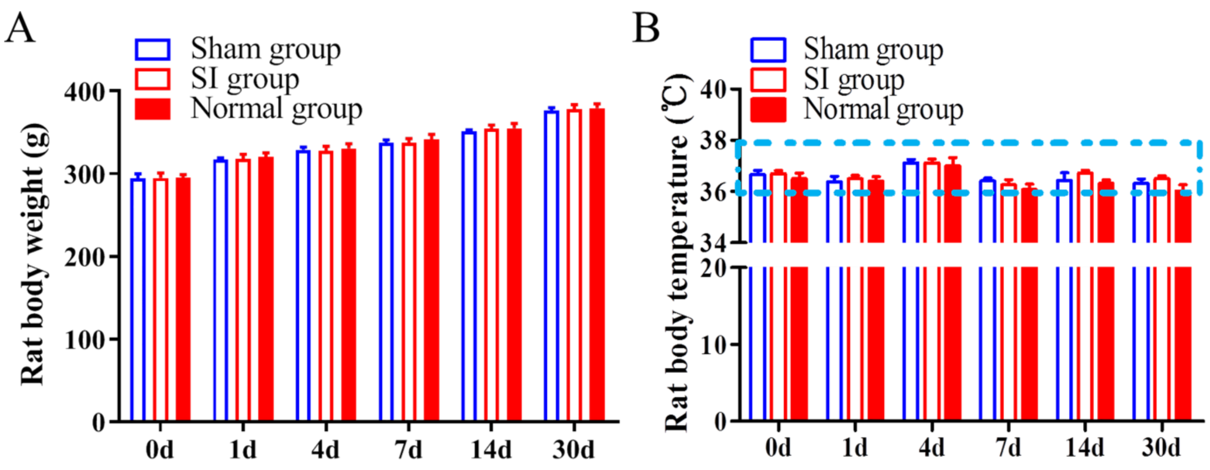

fig. S3. Body weight and body temperature monitoring of rats after SI of nPS and hPS. Rats received subcutaneous implantation (SI) of nPS or hPS with different fiber winding angles (one PS/rat in dorsum) (n=30); rats in the sham group only received surgery to form subcutaneous pockets (one pocket/rat in dorsum) before suture of dorsal skin without PS implantation (n=5); rats in the normal group did not receive any operative procedure (n=5). (A) Body weight of rats in each group increased as normal. (B) Body temperature of rats in each group remained within physiologic temperature range. Dashed lines outline the approximate physiologic temperature range.

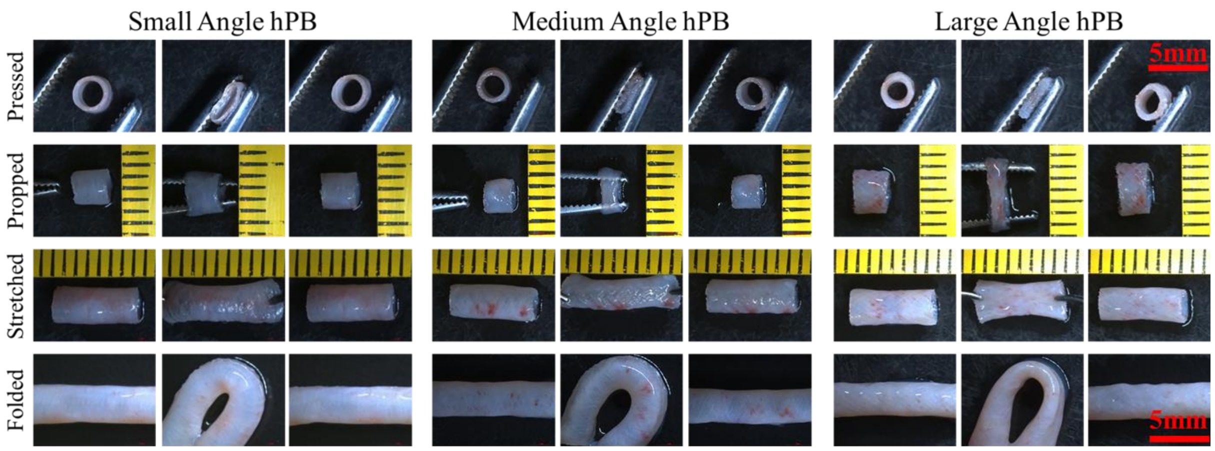

fig. S4. The elastic characterization of hPB. All types of hPB recovered their original shape after being pressed, propped, stretched, or folded. Small angle hPB showed an inferior shape change compared to medium angle hPB (hMPB) and large angle hPB upon being propped. Large angle hPB showed an inferior shape change compared to hMPB and small angle hPB upon being stretched. The fourth row of representative images demonstrate the variable degree of resistance to kink formation of hPB with different fiber winding angles upon being folded at 180°.

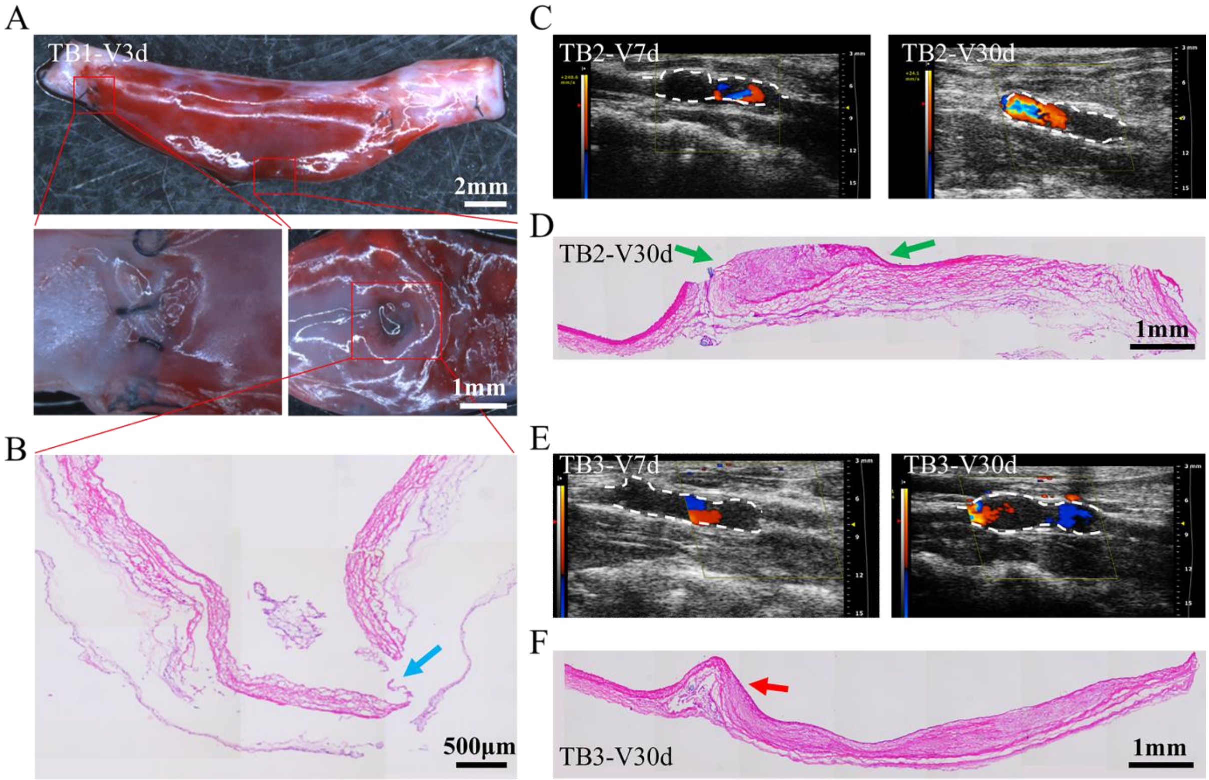

fig. S5. Evaluation of TB as autologous vascular grafts in rAA replacement models. Following traditional biotubes (TB) implantation into rAA (n=3), (A and B) one rat died due to rupture and bleeding at 3 days post-vascular implantation (VI). Stereomicroscopy (A) and H&E (B) images representative of the sizable rupturing of TB, in addition to wrinkling and uneven needle spacing resulting from difficulty of TB handling and suture placement (A, lower panels). (C-F) The other two rats survived for 30 days. Color Doppler ultrasound (CDU) testing showed dilation and blood reflux in the other two TB at 7 and 30 days post-VI (C and E). H&E-stained longitudinal section of the other two TB showed intimal hyperplasia and inversion at the suture site after VI for 30 days (D and F). Blue, green, and red arrows indicate rupture sites, intimal hyperplasia site and suture inversion site, respectively. rAA represents rat abdominal artery; TB-V3d represents TB at 3 days after VI; TB-V30d represents TB at 30 days after VI.

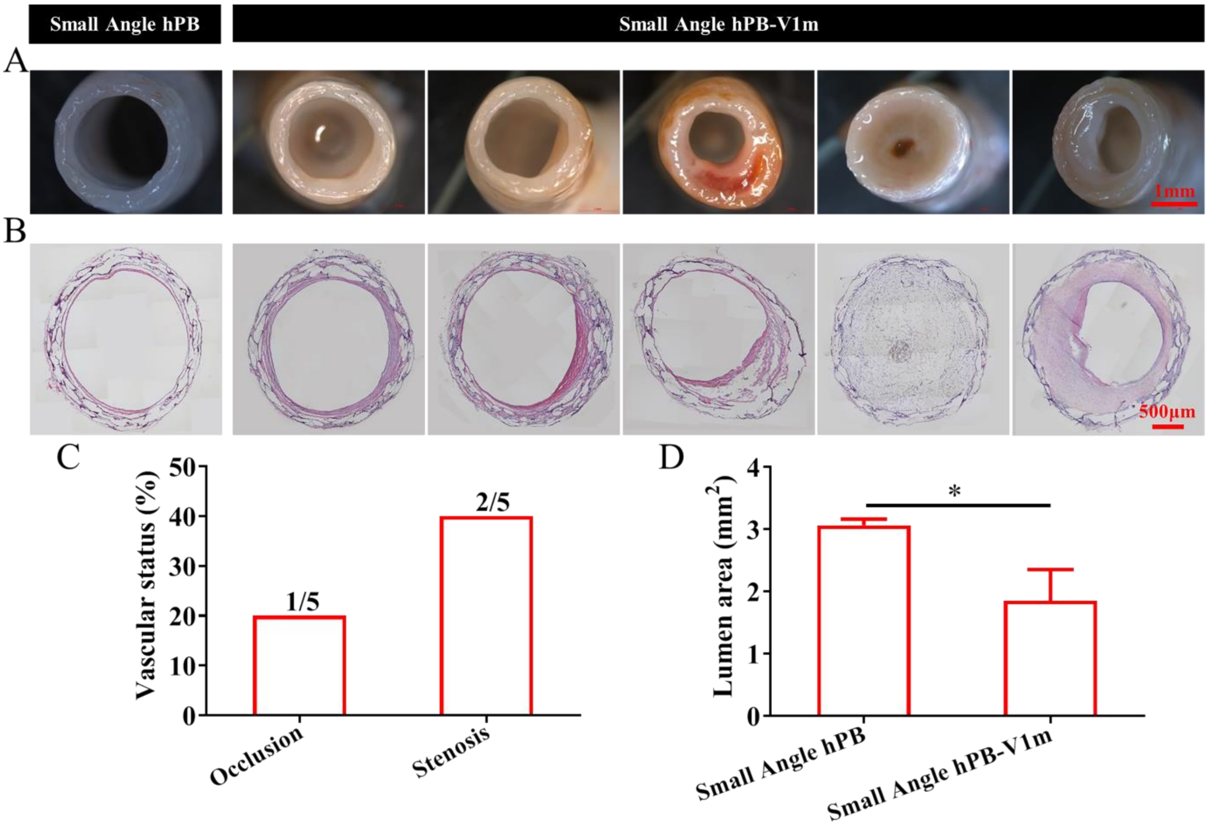

fig. S6. Evaluation of small angle hPB as autologous vascular grafts in a rAA replacement models. (A and B) Stereomicroscopy (A) and H&E (B) images of small angle hPB cross-sections after VI for 1 month (hPB-V1m) showed intimal hyperplasia (IH) compared to small angle hPB before VI (n=5). (C) The occlusion or stenosis rate of small angle hPB-V1m was calculated based on stereomicroscopy images. (D) The lumen area was calculated based on H&E-stained cross-sections. Statistical significance was calculated by a paired Student's t-test. *$P < 0.05$.

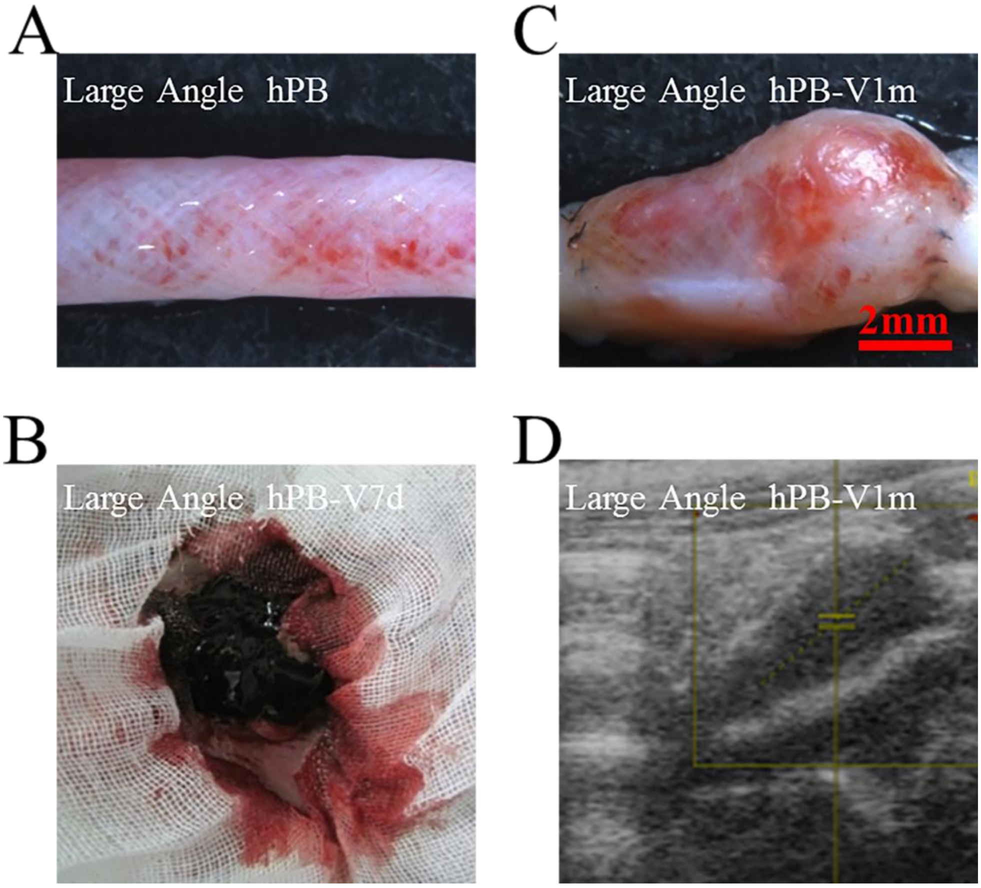

fig. S7. Evaluation of large angle hPB as autologous vascular graft in rAA replacement models. (A) Large angle hPB showed a uniform tubular structure before VI. (B) After VI, four of the five rats died due to large angle hPB rupture, which occurred within the first 3-7 days post-VI. (C and D) Only one rat survived for 1 month, but both stereomicroscopy images (C) and CDU (D) showed large angle hPB dilation. Large angle hPB-V7d represents large angle hPB assessed at 7 days post-VI (n=5); large angle hPB-V1m represents large angle hPB assessed 1 month post-VI (n=5).

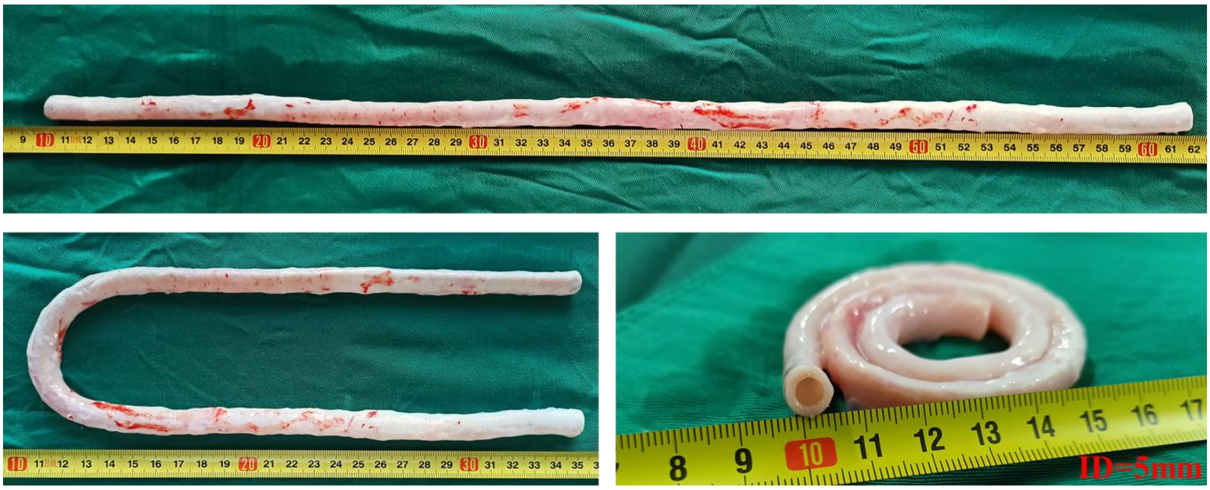

fig. S8. Photographs of hMPB over 50 cm length prepared in sheep dorsal subcutis after SI for 30 days.

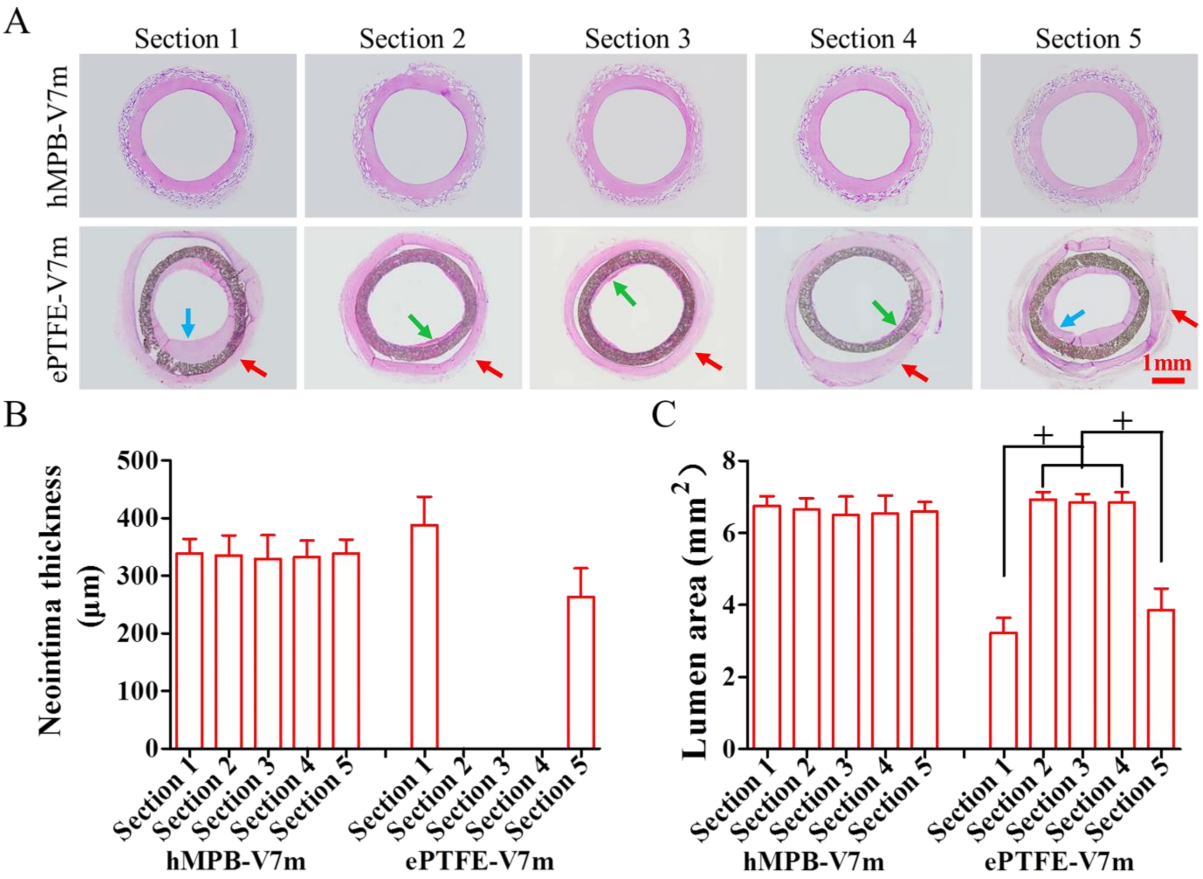

fig. S9. Macroscopic view of H&E-stained cross-sections of hMPB, hMPB-V7m, and ePTFE-V7m after implantation in cCA replacement models. (A) hMPB formed homogeneous neointima without thrombi and fibrous capsule formation, while ePTFE grafts showed obvious IH at both suture ends, thrombi formation and serious fibrous capsule at 7-month post-VI (n=3). (B and C) Quantitative analysis of neointima thickness and lumen area of based on H&E staining cross-section. hMPB-V7m represents hMPB at 7-month post-VI (n=3), ePTFE-V7m represents ePTFE grafts at 7-month post-VI (n=3). cCA represents canine carotid artery (n=3). Red arrows indicate the formation of fibrous capsules. Blue arrows indicate IH. Green arrows indicate thrombi formation. Statistical significance was calculated by two-way ANOVA with Tukey's test, $^{+}P < 0.05$ denotes statistical significance.

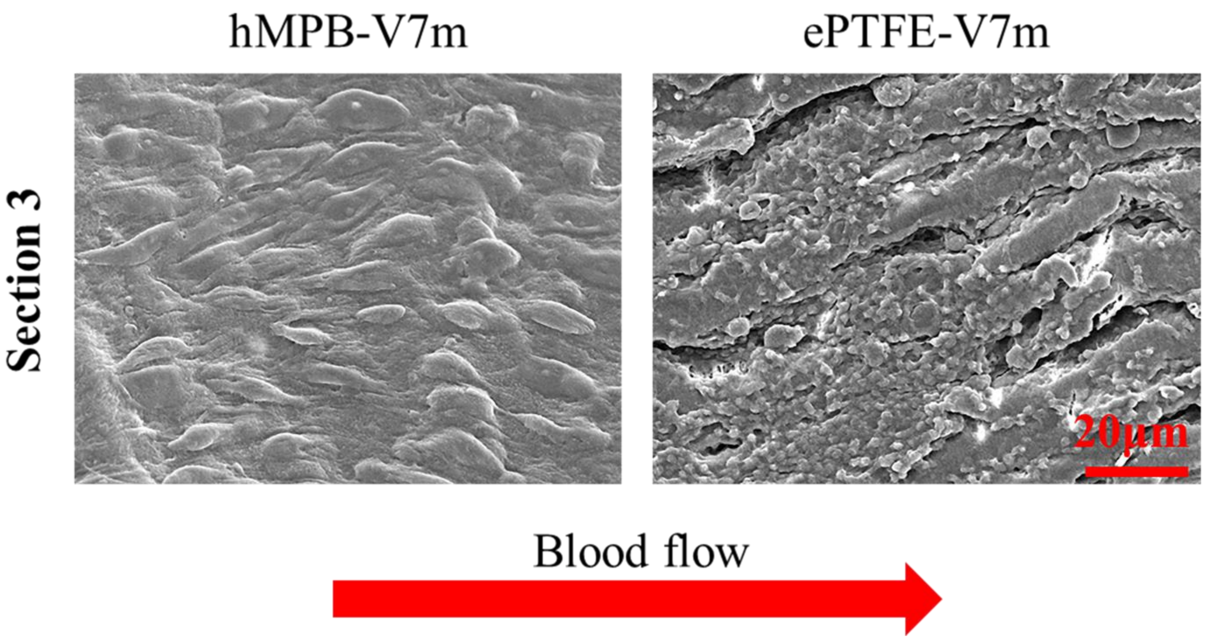

fig. S10. SEM observation of hMPB and ePTFE lumen surfaces after VI for 7 months in cCA replacement model. SEM images showed that the lumen of hMPB-V7m was covered by ECs with cobblestone-like morphology, elongated in the direction of blood flow, whereas a large amount of coagulation matrix components were adhered on the lumen surface of ePTFE-V7m.

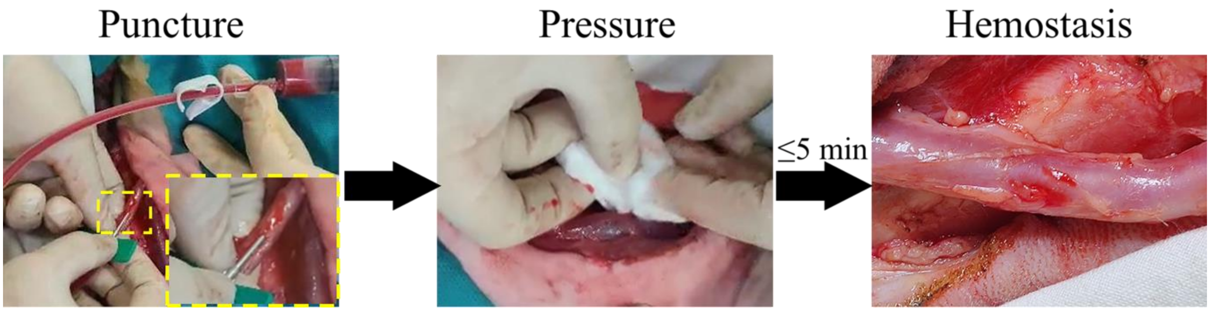

fig. S11. Testing immediate puncture of hMPB segment separated from sheep subcutaneous tissue. Representative photographs showed that after hMPB were auto-grafted as AVG between carotid artery and jugular vein, the segment of hMPB separated from sheep subcutaneous tissue allowed for immediate puncture using a 16G hemodialysis needle and hemostasis was achieved by pressing with medical cotton for 5 min.

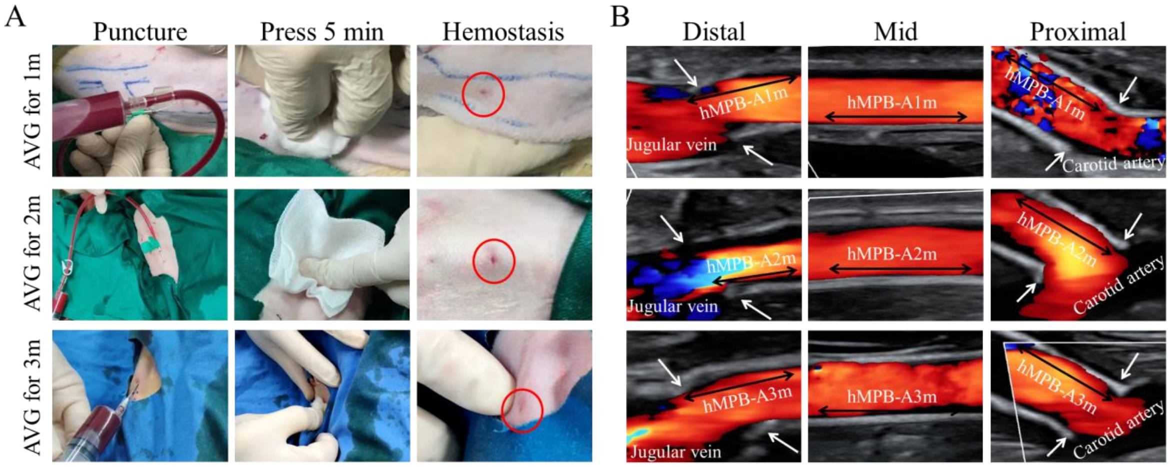

fig. S12. Puncture testing and ultrasound images of hMPB-AVG in sheep models at 1, 2 and 3 months. (A) After puncture using a 16G hemodialysis needle, hemostasis could be achieved by pressing for (at most) 5 min in all hMPBs after every puncture. (B) Representative ultrasound images of hMPB as AVG grafting between the carotid artery and jugular vein in sheep at 1, 2 and 3 months. Red circles indicate puncture sites. White arrows indicated suture sites; hMPB-A1m represents hMPB as AVG in sheep model at 1 month; hMPB-A2m represents hMPB as AVG in sheep model at 2 months; hMPB-A3m represents hMPB as AVG in sheep model at 3 months.

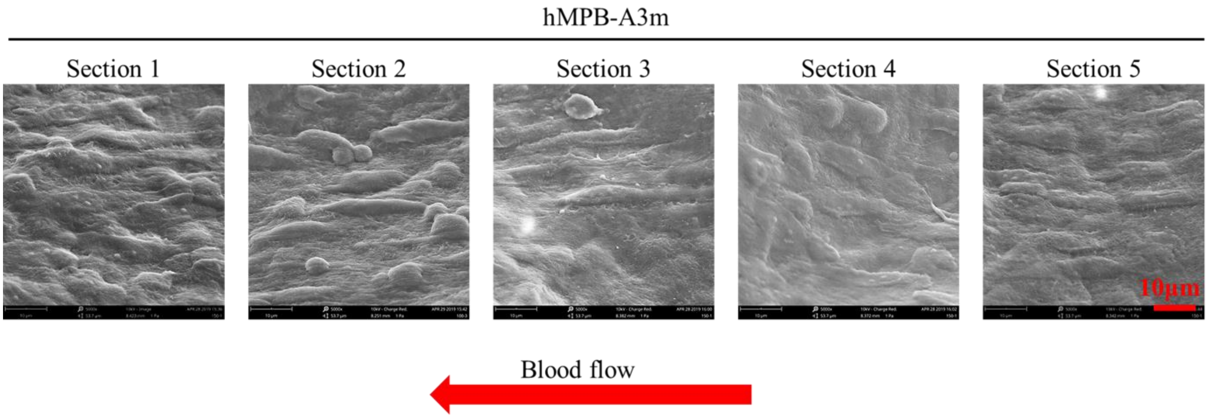

fig. S13. SEM observation of lumen surfaces of hMPB AVG after 3 months in sheep models. SEM images showed that all lumen of hMPB-A3m has been covered by ECs with cobblestone-like morphology and elongated in the direction of blood flow.

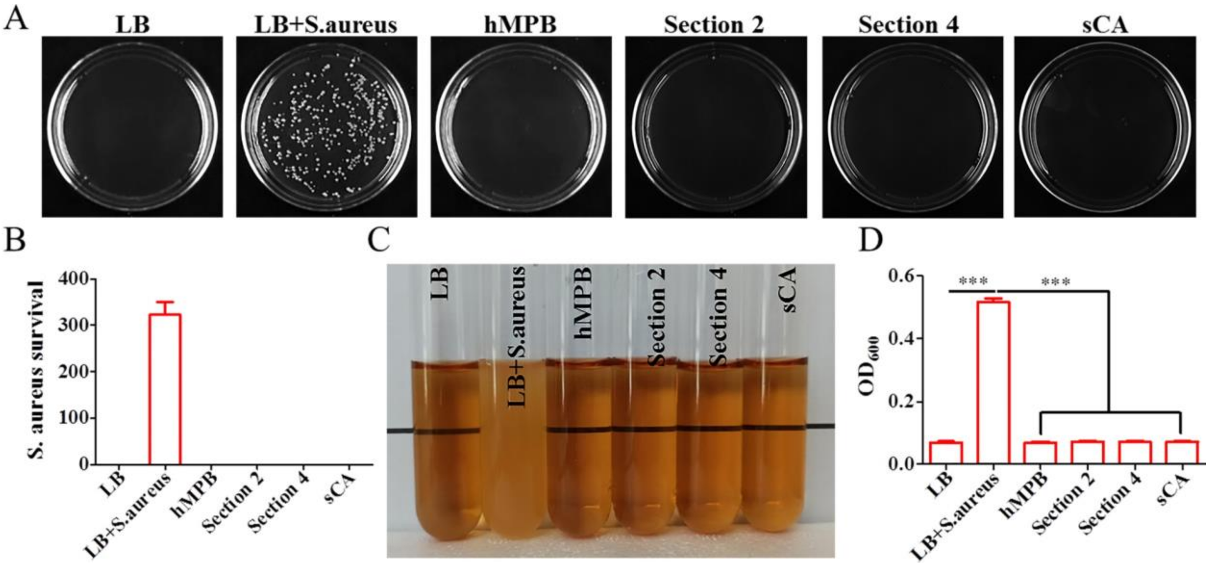

fig. S14. Detection of bacterial infection in AVG explants. (A) Images of actual bacterial CFUs on agar plates obtained from the homogenate of different tissue samples. (B) Colony densities of *S. aureus* in different plates (n=3). (C) Photographs of homogenate added to LB medium and incubated at 37℃ for 8 h. (D) Measurement of the absorbance value at 600 nm to show the amount of bacteria (n=3). Statistical significance was calculated by one-way ANOVA with Tukey's test. ***$P < 0.001$.

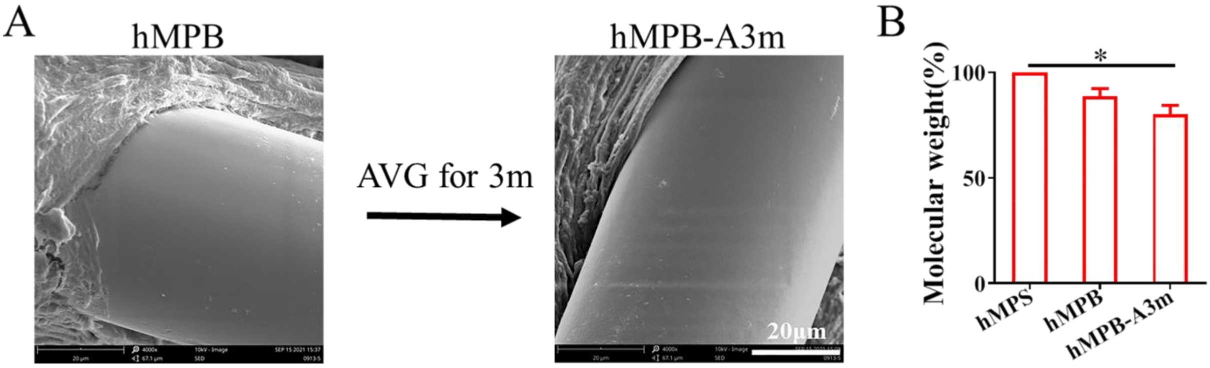

fig. S15 PCL fiber degradation of hMPB AVG implants in sheep models. (A) SEM images showed that the fiber morphology of heat treated medium-fiber-angle PS (hMPS) within hMPB-A3m had no obvious fracturing, or physical changes before and after AVG for 3 months. (B) Molecular weights analysis of hMPS and PCL from extracts of hMPB and hMPB-A3m by Gel Permeation Chromatography (GPC) (n=3). Statistical significance was calculated by one-way ANOVA with Tukey's test. *$P < 0.05$.

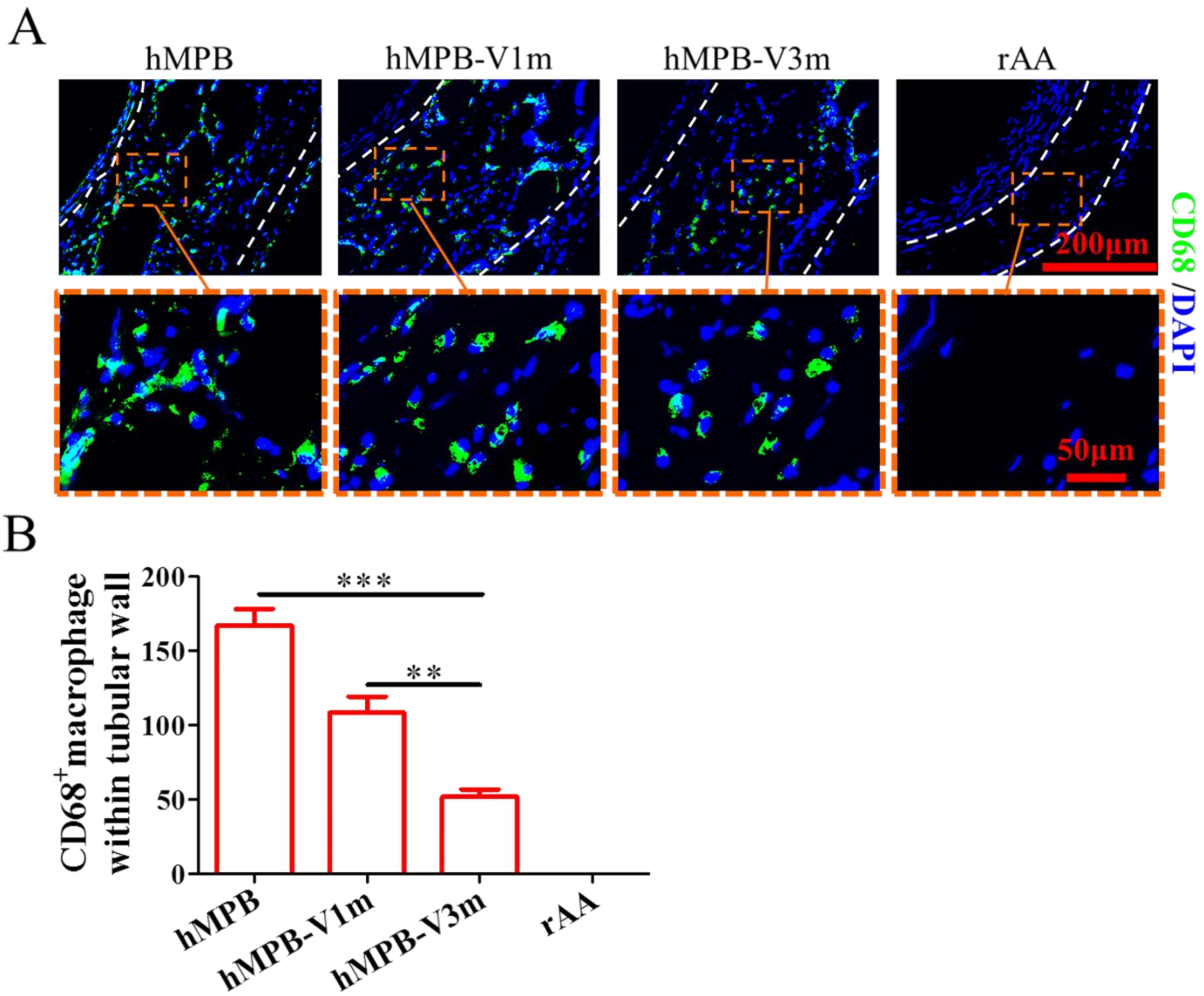

fig. S16. Macrophage infiltration into hMPB after VI for 1 and 3 months in rAA replacement models. (A) IF microscopy images and zoomed insets of sample cross-sections stained for the macrophage marker CD68 and DAPI nuclear stain. (B) The amount of macrophage infiltration within tubular wall (white dashed outlines) was calculated based on low-magnification (upper row) of CD68 stained sections (n=5). Statistical significance was calculated by one-way ANOVA with Tukey's test. $^{**}P < 0.01$, $^{***}P < 0.001$.

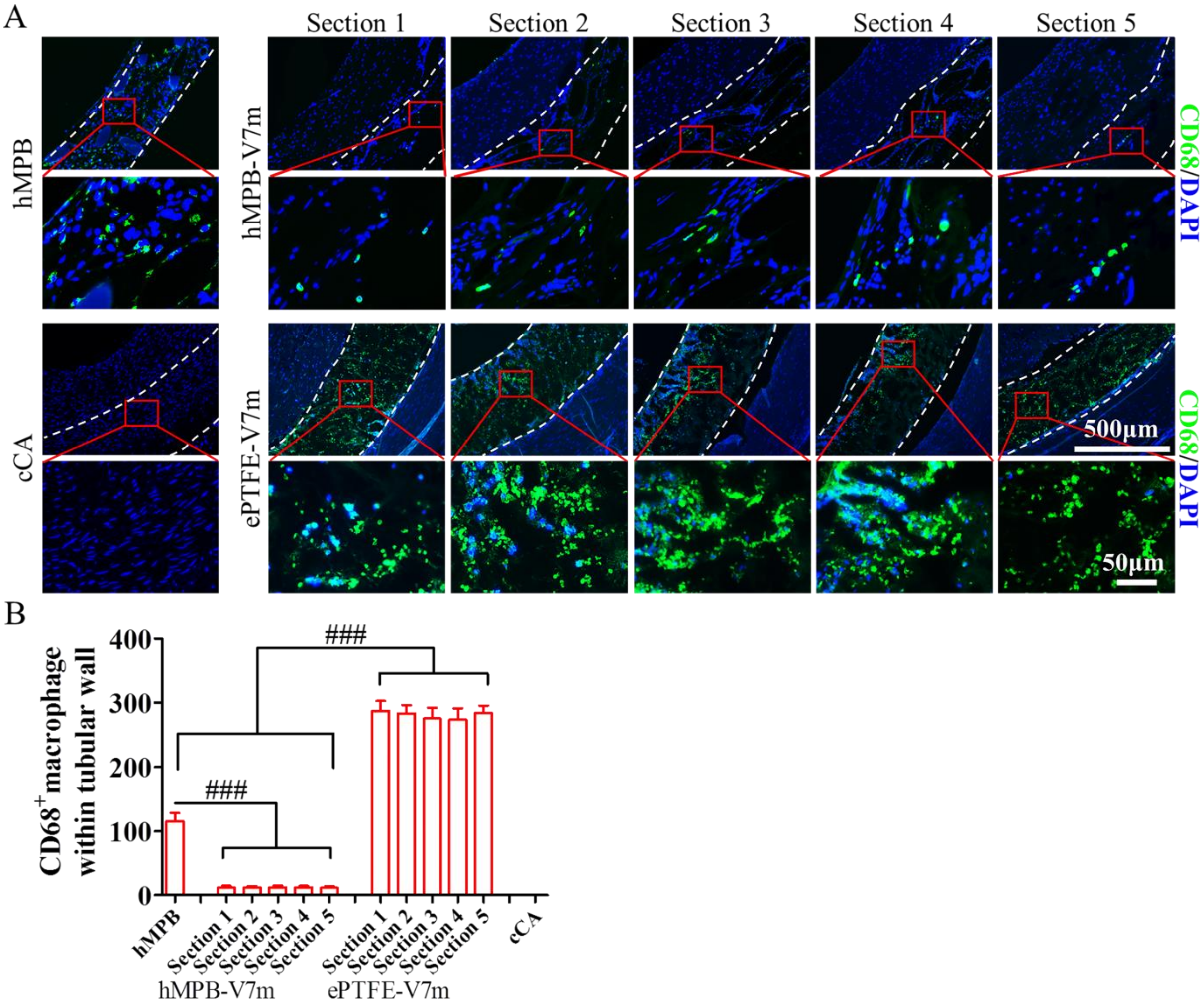

fig. S17. Macrophage infiltration into hMPB and ePFTE grafts after VI for 7 months in cCA replacement models. (A) IF microscopy images and zoomed insets of sample cross-sections stained for the macrophage marker CD68 and DAPI nuclear stain. In hMPB and hMPB-V7m groups, the white dashed outlines mark the hMPS field; in the ePTFE-V7m group, the white dashed outlines mark the ePTFE grafts wall; in the cCA group, white dashed outlines mark the adventitia field. (B) The amount of macrophage infiltration within tubular walls (white dashed outlines) were calculated based on low-magnification (upper rows) CD68 stained sections (n=3). Statistical significance was calculated by two-way ANOVA with Tukey's test. $^{###}P < 0.001$ indicates statistical significance.

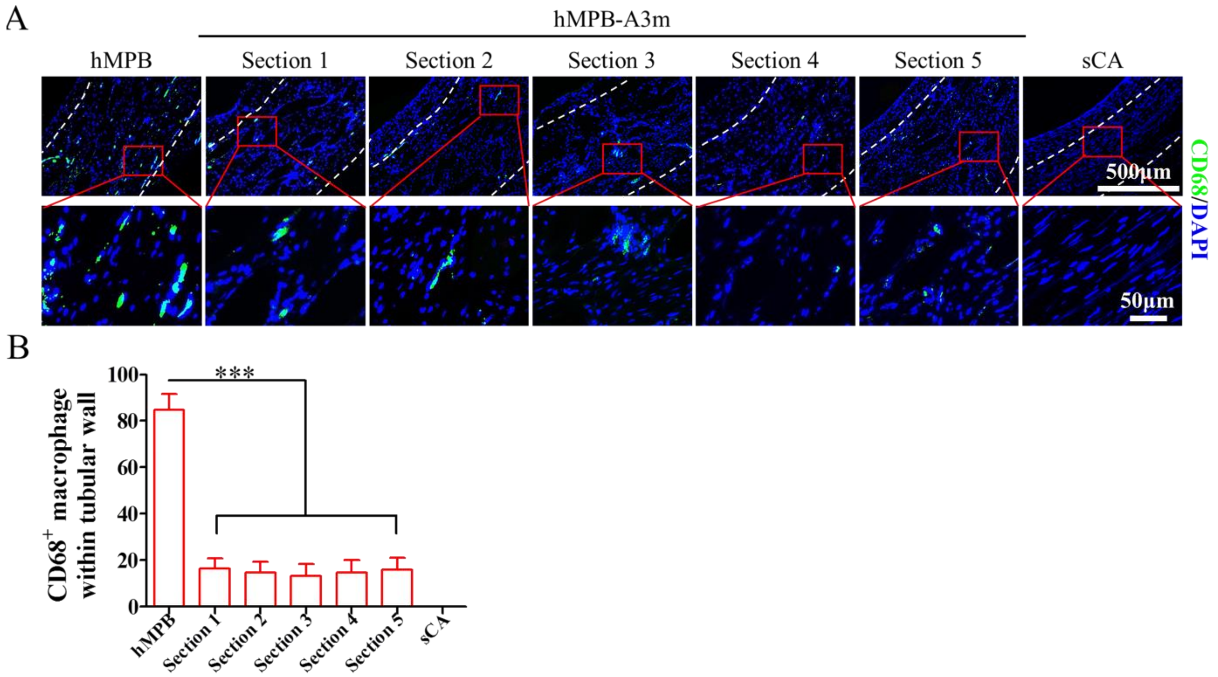

fig. S18. Macrophage infiltration into hMPB AVG after 3 months grafting in sheep models. (A) IF microscopy images and zoomed insets of sample cross-sections stained for the macrophage marker CD68 and DAPI nuclear stain. In hMPB and hMPB-A3m groups, white dashed outlines mark the hMPS field**.** In the sCA group, white dashed outlines mark the adventitia field. (B) The amount of macrophage infiltration within tubular walls (white dashed outlines) were calculated based on low-magnification (upper row) CD68 stained sections (n=3). Statistical significance was calculated by one-way ANOVA with Tukey's test. $^{***}P < 0.001$.

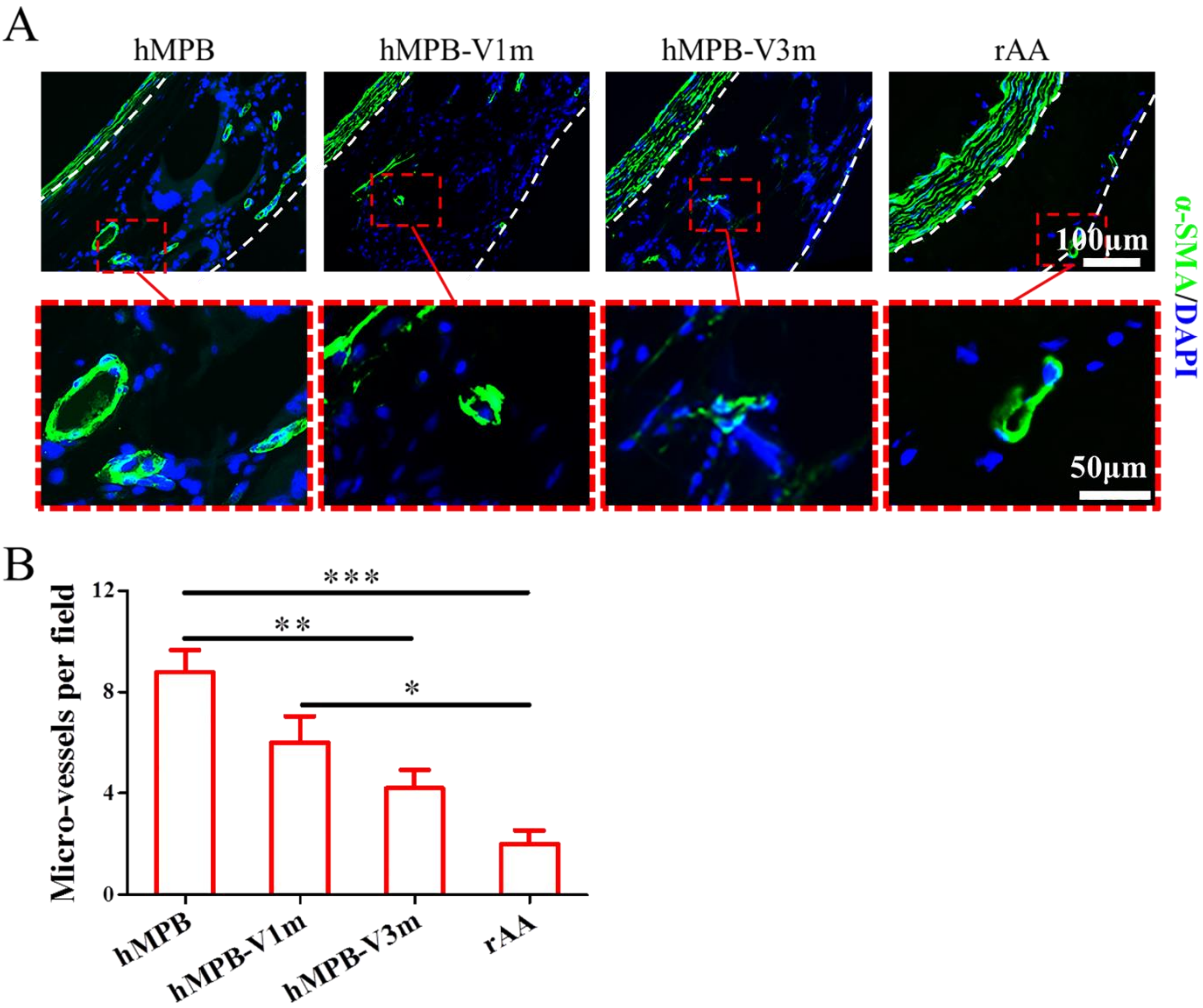

fig. S19. IF staining of α-SMA in hMPB before and after VI for 1 and 3 months in rAA replacement models. (A) Upper row of IF staining shows the distribution of α-SMA^{+} cells and micro-vessels. White dashed outlines approximate hMPS field in hMPB, hMPB-V1m and hMPB-V3m. In rAA, white dashed outlines indicate the adventitia boundaries. Lower row images represent the high magnification areas indicates by red dashed line boxes. hMPB-V1m represents hMPB at 1 month after VI; hMPB-V3m represents hMPB at 3 months after VI. (B) The micro-vessels per field were counted based on α-SMA/DAPI upper row of images (n=5). Statistical significance was calculated by one-way ANOVA with Tukey's test. *$P < 0.05$, **$P < 0.01$ and ***$P < 0.001$.

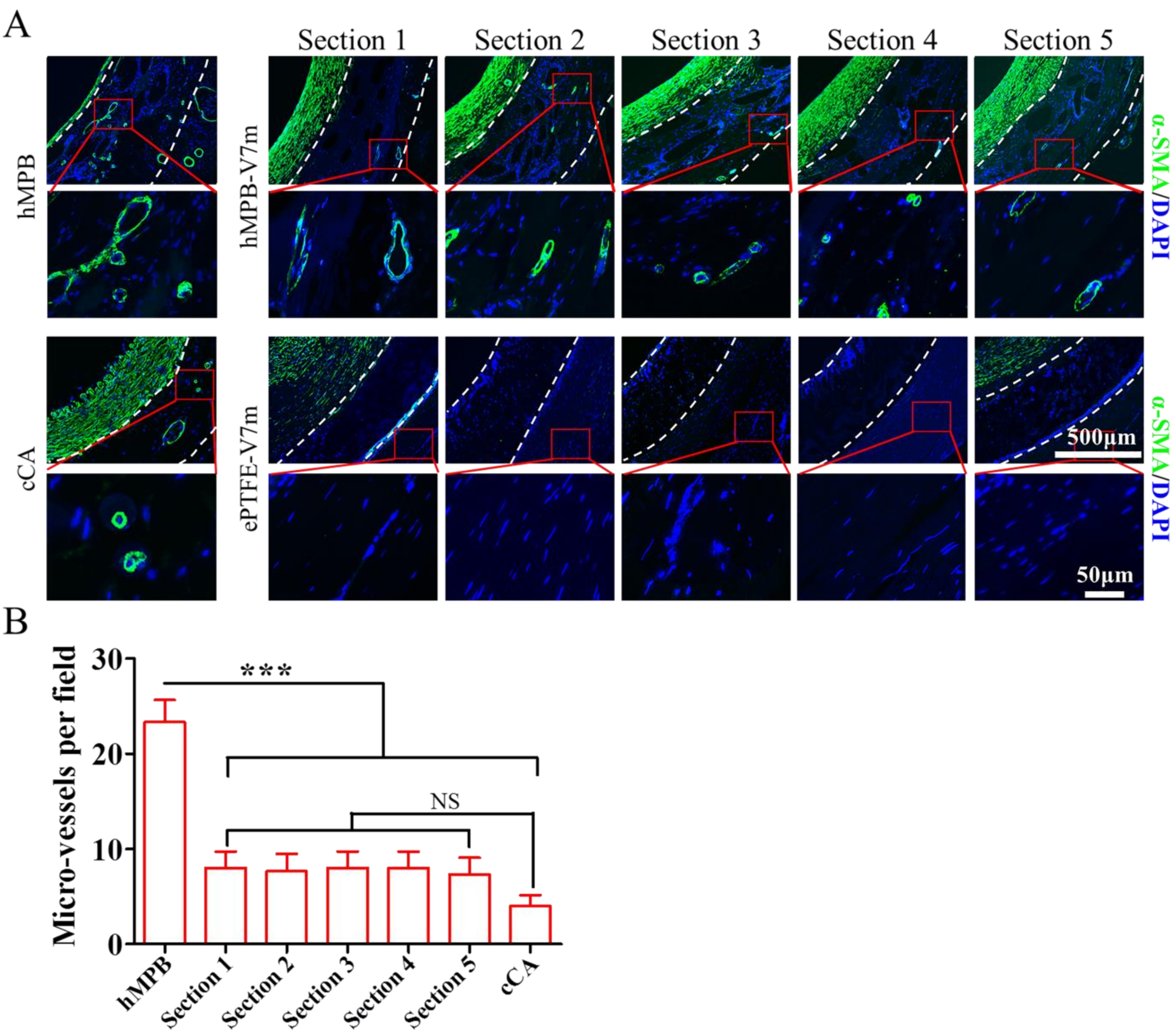

fig. S20. Vascularization of hMPB and ePFTE grafts after VI for 7 months in cCA replacement models. (A) IF microscopy images and zoomed insets of sample cross-sections stained for α-SMA antibody and DAPI. In hMPB and hMPB-V7m group, white dashed outlines the hMPS field**.** In the ePTFE-V7m group, white dashed outlines mark the ePTFE graft walls**.** In the cCA group, white dashed outlines mark the adventitia field. (B) The number of micro-vessels per field was calculated based on the whole low-magnification (upper rows) α-SMA stained sections (n=3). Statistical significance was calculated by one-way ANOVA with Tukey's test. $***P < 0.001$. NS indicates not significant.

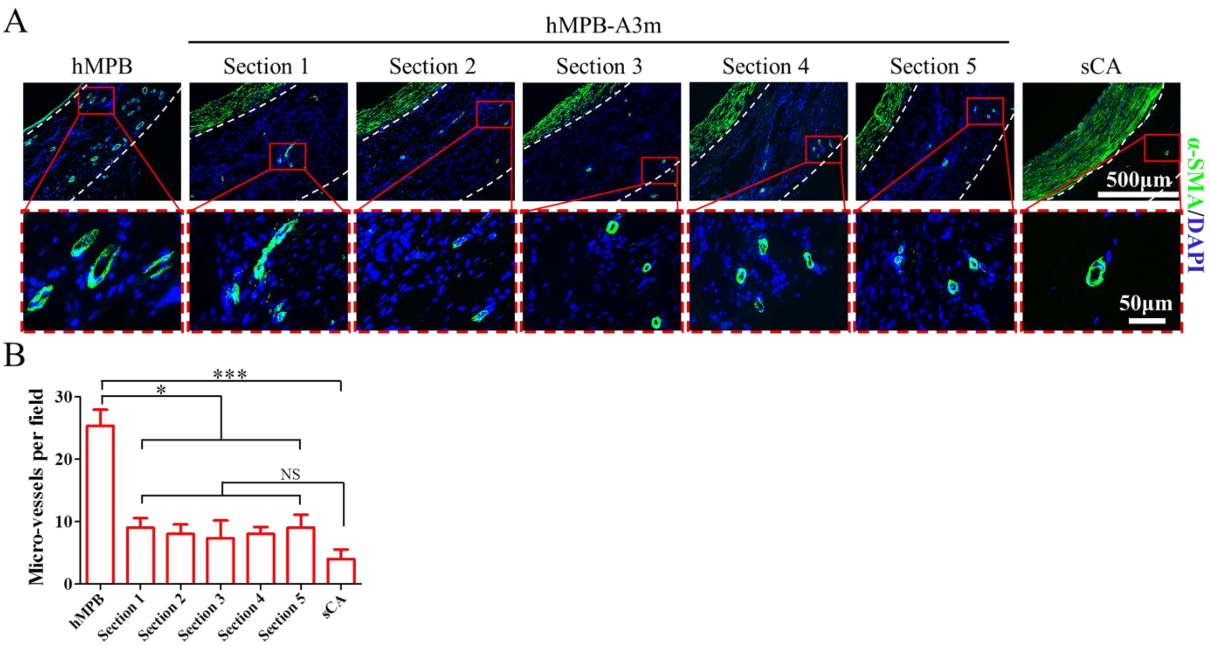

fig. S21. Vascularization of hMPB AVG after grafting for 3 months in sheep models. (A) IF microscopy images and zoomed insets of sample cross-sections stained for α-SMA antibody and DAPI nuclear stain. Red boxed images indicate the micro-vessels. In hMPB and hMPB-A3m groups, white dashed outlines mark the hMPS field**.** In the sCA group, white dashed outlines indicate the adventitia field. (B) The number of micro-vessels per field was calculated based on low-magnification (upper row) α-SMA/DAPI stained sections (n=3). Statistical significance was calculated by one-way ANOVA with Tukey's test. *$P < 0.05$, ***$P < 0.001$. NS indicates not significant.

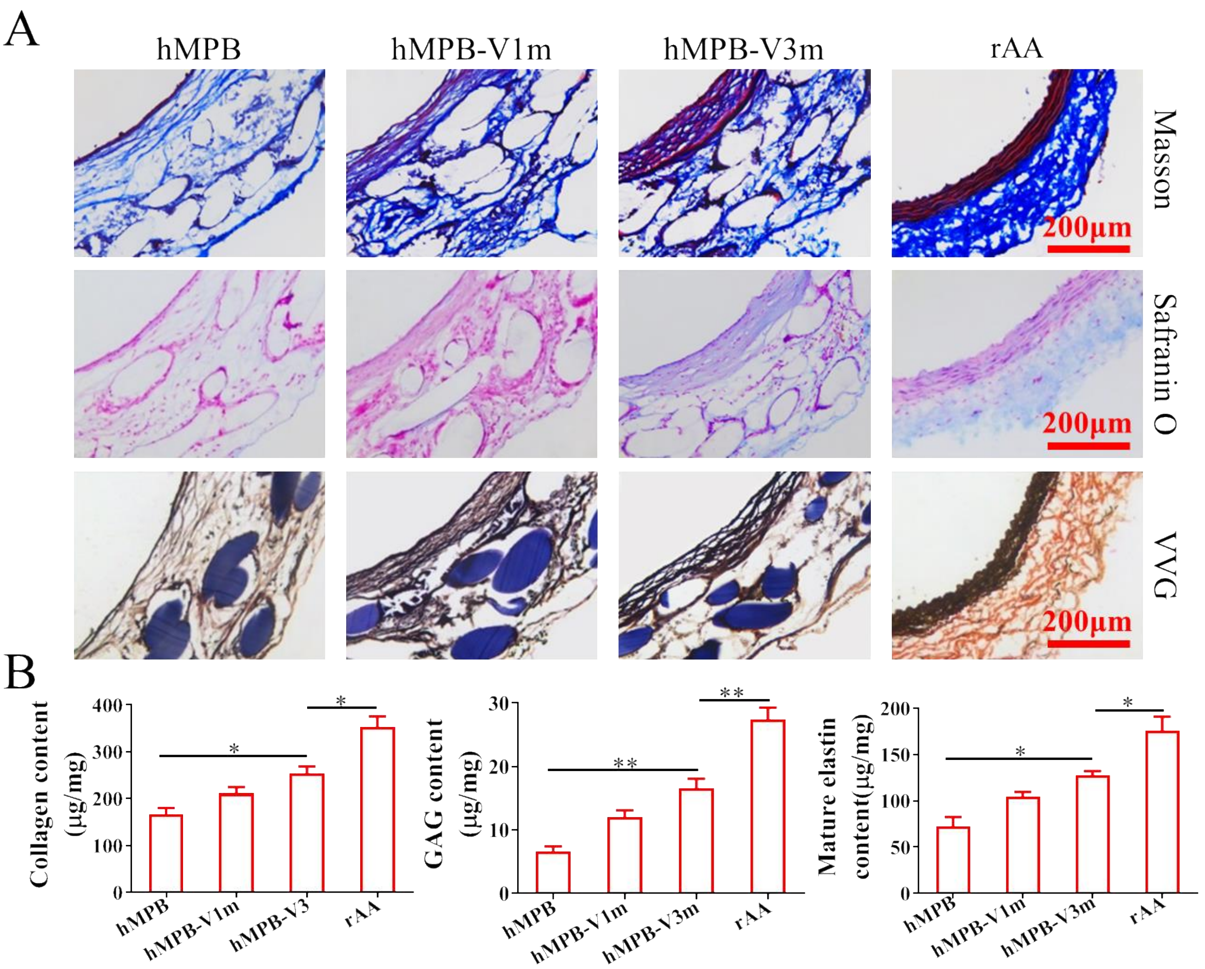

fig. S22. ECM remodeling of hMPB before and after VI for 1 and 3 months in rAA replacement models. (A) Collagen (Masson's Trichrome), GAG (Safranin O), and elastin (VVG) were visualized. (B) Quantification of total collagen, GAG and mature elastin (n = 3). The rAA as control were also analyzed by the same protocol. Statistical significance was calculated by one-way ANOVA with Tukey's test. *P < 0.05, **P < 0.01. ECM represents extracellular matrix.

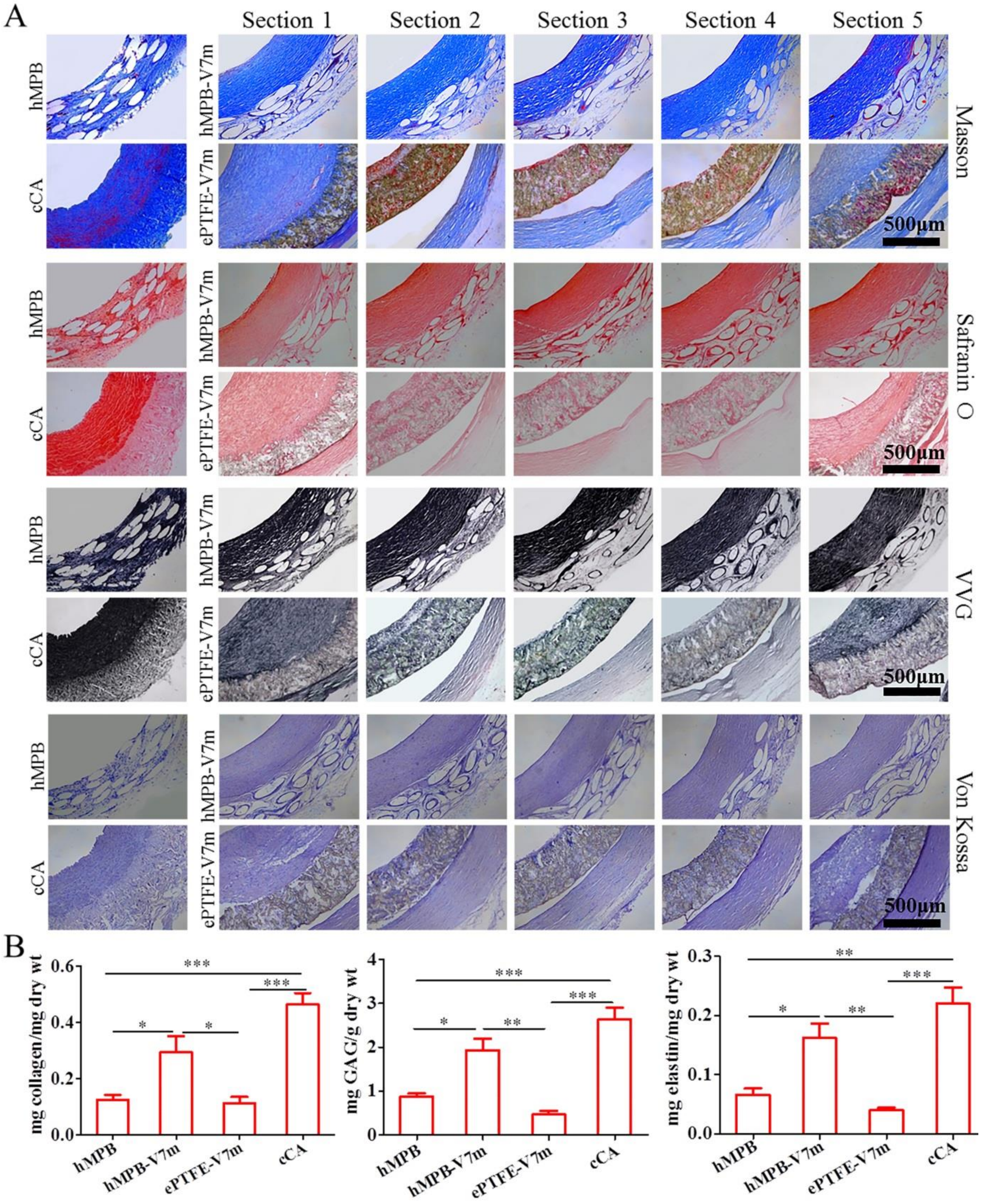

fig. S23. ECM remodeling of hMPB and ePFTE grafts after VI for 7 months in cCA replacement models. (A) Collagen (Masson's Trichrome), GAG (Safranin O), elastin (VVG), and calcification (Von Kossa) were visualized. (B) Quantification of total collagen, GAG and mature elastin (n = 3). The cCA and hMPB as control were also analyzed by the

same protocol. Statistical significance was calculated by one-way ANOVA with Tukey's test. *$P < 0.05$, **$P < 0.01$, ***$P < 0.001$.

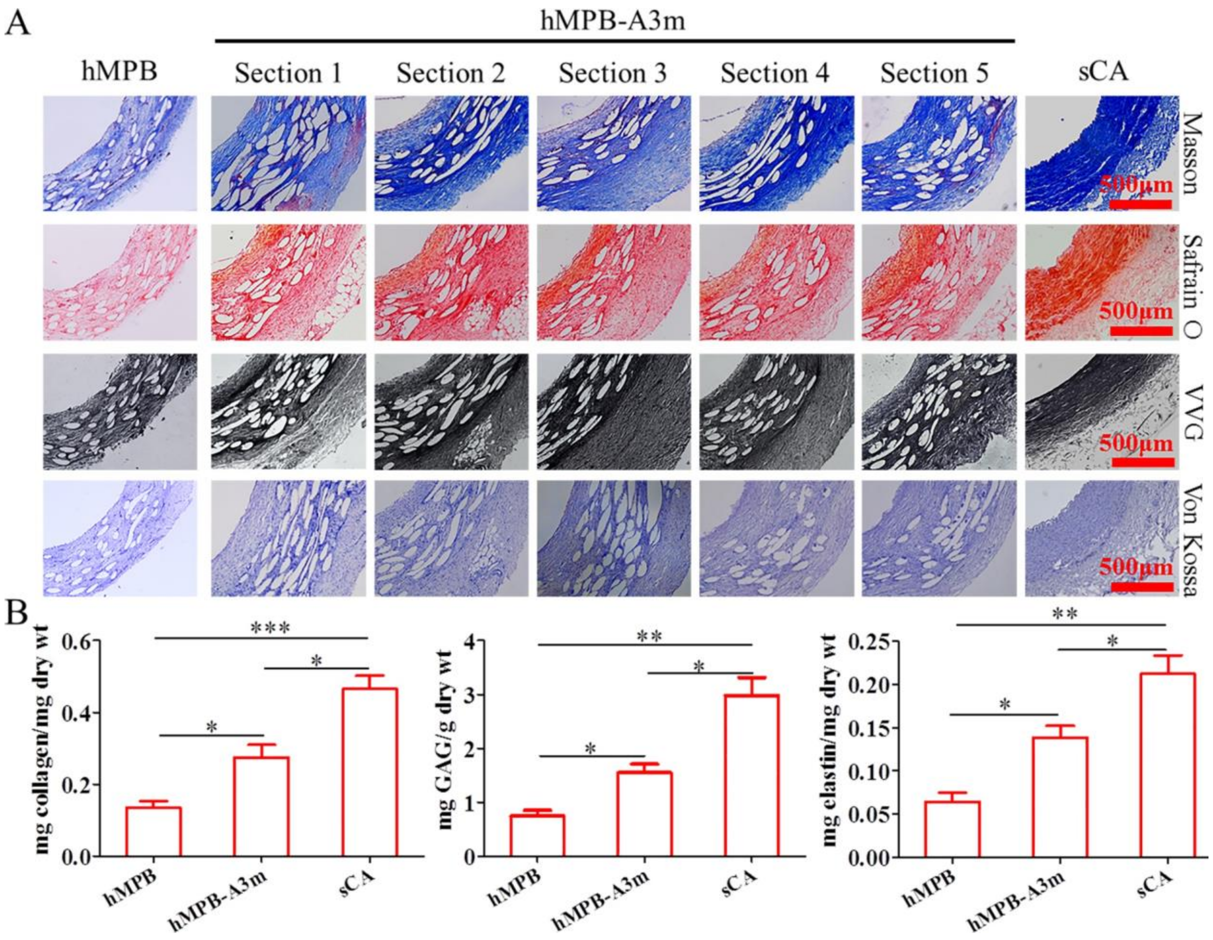

fig. S24. ECM remodeling of hMPB AVG after grafting for 3 months in sheep models. (A) Collagen (Masson's Trichrome), GAG (Safranin O), elastin (VVG), and calcification (Von Kossa) were visualized. (B) Quantification of total collagen, GAG and mature elastin (n=3). sCA and hMPB as control were also analyzed by the same protocol. Statistical significance was calculated by one-way ANOVA with Tukey's test. *$P < 0.05$, **$P < 0.01$, ***$P < 0.001$.

table S1. List of abbreviations (in order of appearance).

Abbreviation	Definition
PCL	poly(ε-caprolactone)
PS	PCL fiber skeletons
PB	PS-reinforced biotubes
hMPB	heat-treated medium-fiber-angle PB
AVG	arteriovenous graft
ePTFE	expanded polytetrafluoroethylene
IH	intimal hyperplasia
TEVGs	Tissue-engineered vascular grafts
EC	endothelial cell
FBR	foreign body response
TB	traditional biotubes
ECM	extracellular matrix
nPS	non-heat treated PS
hPS	heat treated PS
SEM	scanning electron microscopy
SI	subcutaneous implantation
hMPS	heat-treated medium angle PS
H&E	haematoxylin and eosin
nPB	nPS-reinforced biotubes
hPB	hPS-reinforced biotubes
rAA	rat abdominal artery
VI	vascular implantation
CDU	Color Doppler ultrasound
hMPB-V1m	hMPB after VI for 1 month
hMPB-V3m	hMPB after VI for 3 month
eNOS	endothelial nitric oxide synthase
MYH	myosin heavy chain I
IF	immunofluorescence
SMCs	smooth muscle cells
KCl	potassium chloride
AD	adrenaline
ACh	acetylcholine
SNP	sodium nitroprusside
α-SMA	alpha smooth muscle actin
cCA	canine carotid artery
CT	computed tomography
MRI	magnetic resonance imaging
hMPB-V7m	hMPB at 7-month post-VI
ePTFE-V7m	ePTFE grafts at 7-month post-VI
vWF	Von Willebrand Factor
IHC	immunohistochemistry
WB	Western blot
GPC	Gel Permeation Chromatography
sCA	sheep carotid artery
hMPB-A3m	hMPB after 3-month post-AVG
CNN	calponin
GAG	glycosaminoglycan
VVG	Verhoeff–van Gieson

table S2. Preparation parameters and morphological characterization of PS.

Outer diameter of silicon tube (mm)	Melt spinning parameters				Non-heat treatment				Heat treatment			
	Feeding rate (mL/h)	Lateral translation speed (mm/s)	The number of lateral translations (round trips)	Rotation speeds (rpm)	Fiber winding angle (°)	Fiber diameter (μm)	Pore size (μm)	Thickness of PCL fiber skeleton (μm)	Fiber winding Angle (°)	Fiber diameter (μm)	Pore size (μm)	Thickness of PCL fiber skeleton (μm)
2	0.50	5.00	30	300	30.48± 1.48	60.20± 1.50	108.10± 4.72	353.90± 16.70	31.19± 1.58	59.31± 1.01	111.60± 7.28	350.50± 7.53
2	0.50	10.00	45	300	51.01± 2.36	59.37± 1.91	188.80± 7.50	354.90± 15.93	51.14± 2.37	59.93± 1.49	190.30± 5.00	349.90± 9.02
2	0.50	20.00	120	197	111.50± 2.77	60.25± 1.79	261.40± 9.75	354.10± 15.88	112.20± 2.56	60.36± 1.74	261.2± 11.31	351.10± 11.09
3	0.60	28.00	50	260	50.48± 2.91	59.28± 1.75	190.40± 8.61	371.40± 20.12	51.21± 2.08	60.40± 1.61	189.60± 8.91	375.45± 19.21
5	0.90	36.00	75	180	50.27± 2.66	59.71± 1.64	189.7± 8.14	499.71± 18.24	49.86± 3.14	59.37± 1.56	190.70± 7.46	502.46± 19.67
